# MZ1, a BRD4 inhibitor, exerted its anti-cancer effects by suppressing SDC1 in glioblastoma

**DOI:** 10.1186/s12885-024-11966-8

**Published:** 2024-02-16

**Authors:** Gen Li, Liya Ma, Chenxi Feng, Hongli Yin, Jianping Bao, Di Wu, Zimu Zhang, Xiaolu Li, Zhiheng Li, Chun Yang, Hairong Wang, Fang Fang, Xiaohan Hu, Mei Li, Lixiao Xu, Yunyun Xu, Hansi Liang, Tianquan Yang, Jianwei Wang, Jian Pan

**Affiliations:** 1 Institute of Pediatric Research, Children’s Hospital of Soochow University, Suzhou, 215025 P.R. China; 2 College of Basic Medicine and Forensic Medicine, Henan University of Science and Technology, Luoyang, 471023 P.R. China; 3 Department of Neonatology, Children’s Hospital of Soochow University, Suzhou, 215025 P.R. China; 4 Jiangsu Institute of Clinical Immunology, The First Affiliated Hospital of Soochow University, Suzhou, 215006 P.R. China; 5 Department of Neurosurgery, Children’s Hospital of Soochow University, Suzhou, 215025 P.R. China

**Keywords:** GBM, BRD4, SEs, PROTAC, SDC1

## Abstract

**Background:**

Glioblastoma (GBM) is a relatively prevalent primary tumor of the central nervous system in children, characterized by its high malignancy and mortality rates, along with the intricate challenges of achieving complete surgical resection. Recently, an increasing number of studies have focused on the crucial role of super-enhancers (SEs) in the occurrence and development of GBM. This study embarks on the task of evaluating the effectiveness of MZ1, an inhibitor of BRD4 meticulously designed to specifically target SEs, within the intricate framework of GBM.

**Methods:**

The clinical data of GBM patients was sourced from the Chinese Glioma Genome Atlas (CGGA) and the Gene Expression Profiling Interactive Analysis 2 (GEPIA2), and the gene expression data of tumor cell lines was derived from the Cancer Cell Line Encyclopedia (CCLE). The impact of MZ1 on GBM was assessed through CCK-8, colony formation assays, EdU incorporation analysis, flow cytometry, and xenograft mouse models. The underlying mechanism was investigated through RNA-seq and ChIP-seq analyses.

**Results:**

In this investigation, we made a noteworthy observation that MZ1 exhibited a substantial reduction in the proliferation of GBM cells by effectively degrading BRD4. Additionally, MZ1 displayed a notable capability in inducing significant cell cycle arrest and apoptosis in GBM cells. These findings were in line with our in vitro outcomes. Notably, MZ1 administration resulted in a remarkable decrease in tumor size within the xenograft model with diminished toxicity. Furthermore, on a mechanistic level, the administration of MZ1 resulted in a significant suppression of pivotal genes closely associated with cell cycle regulation and epithelial-mesenchymal transition (EMT). Interestingly, our analysis of RNA-seq and ChIP-seq data unveiled the discovery of a novel prospective oncogene, SDC1, which assumed a pivotal role in the tumorigenesis and progression of GBM.

**Conclusion:**

In summary, our findings revealed that MZ1 effectively disrupted the aberrant transcriptional regulation of oncogenes in GBM by degradation of BRD4. This positions MZ1 as a promising candidate in the realm of therapeutic options for GBM treatment.

**Supplementary Information:**

The online version contains supplementary material available at 10.1186/s12885-024-11966-8.

## Background

Glioblastoma multiforme (GBM), a highly malignant primary tumor arising from neuroglial stem or progenitor cells, has the potential to emerge across all age groups [1]. On average, GBM patients survive for approximately 14 months, and the 5-year survival rates are below 9.8% [2]. Despite the advancement in therapeutic approaches for GBM, including surgical excision, chemotherapy, radiation therapy, or their integration, the outlook remains bleak [3]. Thus, it is imperative to pinpoint innovative chemotherapeutic compounds to enhance the management of GBM in humans.

Previous studies have highlighted that, in comparison to regular somatic cells, cancer cells frequently display heightened levels of overall transcriptional activity and involvement in oncogenic transcription. This heightened condition provides them with amplified opportunities to participate in pathways associated with carcinogenesis [4]. The activation of target genes at the transcriptional level primarily relies on the involvement of specific transcription factors and enhancer elements. Enhancers possess the capability to initiate transcription for genes situated far away in the linear DNA sequence, regardless of their relative position and orientation. Disruptions in the proper functioning of enhancers are frequently linked to the processes of tumor formation and development [5]. Super-enhancers (SEs) are extensive collections of various enhancer-like elements, covering several kilobases in size, and possessing the unique capability to significantly enhance the transcription of their target genes in comparison to conventional enhancers [6].

Prior investigations have unveiled that the abnormal expression of pivotal oncogenes in the majority of pediatric cancers stems from anomalous transcription triggered by SEs. These include the expression of JUN in GBM [7], MYCN in neuroblastoma [8], and GFI1 in medulloblastoma [9]. Moreover, the disturbance of the RET finger protein–histone deacetylase complex influences the condition of H3K27Ac-mediated super-enhancers, leading to heightened susceptibility to TMZ drug treatment and extended survival for GBM patients [10]. Hence, it holds potential benefits to uncover synthetic inhibitors that precisely target SEs for the clinical treatment of GBM.

Presently, among the SE-associated elements identified as targets for cancer therapy, notable candidates include BRD4, CDK7, CDK8, CDK19, and EP300 [11]. SE inhibitors, such as JQ1 [12] and CBP300 [11], possess the ability to impede the phosphorylation of Pol II, decrease H3K27Ac levels, and initiate the breakdown of SEs by obstructing the interaction between SE-associated elements and their specific targets. As a result, this process culminates in a significant decrease in the transcription of oncogenes intricately associated with SEs.

BRD4 belongs to the bromodomain and extra-terminal (BET) family, characterized by its unique capability to precisely recognize and target the acetylated lysine residues on histones [13]. This interaction promotes the recruitment of the positive transcription elongation factor P-TEFb, allowing BRD4 to assume a crucial role in controlling the elongation stage of RNA polymerase II transcription. Notably, this regulatory mechanism is prominently focused on governing genes linked with SEs [14, 15]. BRD4 assumes a crucial role in numerous biological processes, including cell proliferation, immune response, metabolism repair, and embryonic development, due to its essential function in transcriptional regulation [16]. More and more research has demonstrated the upregulation of BRD4 expression in diverse solid tumors, including, prostate cancer [17], gastrointestinal cancer [18], and brain tumors [19]. BRD4 expression is elevated in GBM, exhibiting a converse correlation with GBM prognosis, which emphasizes BRD4’s central role in GBM tumorigenesis [20–23]. In line with this, the inhibition of BRD4 consistently leads to significant suppression of cell growth, invasion, and migration, as well as a restraint on cell cycle progression and inducing cell apoptosis, both in laboratory settings and in live organisms [24, 25]. These outcomes strongly suggest BRD4’s involvement in driving GBM progression, underscoring its promise as a potential therapeutic target for treating GBM.

BRD4 activity can be effectively suppressed through the utilization of inhibitors or degraders. BRD4 inhibitors constitute a category of small-molecule compounds that exhibit the potential to significantly enhance cancer treatment by mimicking the effects of acetyl-lysine [26]. Notable examples include JQ1 [24], I-BET151 [20], and OTX015 [27], which have been extensively investigated as BRD4 inhibitors in numerous preclinical studies focused on GBM. Several recent studies have emphasized that the utilization of BRD4 inhibitors leads to substantial suppression of BRD4 protein. Simultaneously, resistance to these inhibitors has been documented across various tumor types [28, 29]. Collectively, these elements contribute to the constrained effectiveness of BRD4 inhibitors in the realm of cancer therapy. In contrast to BRD4 inhibitors, BRD4 degraders represent chimeric molecules that harness the potential of proteolytic targeting chimera (PROTAC) technology to promote the depletion of BET proteins [30]. Presently, several BRD4 degraders have been created for GBM treatment and have exhibited substantial anti-tumor impacts in GBM models, including GNE987 [23], dBET6 [31], ZBC260 [32], and ARV-825 [33].

MZ1 represents another developed PROTAC composed of the von Hippel-Lindau ligand linked to a BRD4 ligand. MZ1 rapidly and potently induces the degradation of BRD4, with a higher selectivity compared to BRD2 and BRD3 [34, 35]. Nevertheless, as of now, there has been no assessment of MZ1’s functionality within GBM models. In this current study, our objective is to investigate the impact of MZ1 on GBM cell lines and xenograft mouse models through a series of in vitro and in vivo functional experiments.

## Methods

### Cell culture, antibodies, and chemicals

The human GBM cell lines U87, A172, LN229, and U251 were purchased from the ATCC (USA). The cells were grown in Dulbecco’s Modified Eagle’s Medium with 10% fetal bovine serum, 100 units/ml streptomycin, and 100 μg/ml penicillin. The primary antibodies against BRD4 (13440S), BRD2 (5848S), PARP (9542S), VHL (68547S), GAPDH (5174S) were purchased from Cell Signaling Technology (USA), Ki-67 (ab16667), PCNA (ab18197) were purchased from Abcam (USA), and SDC1 (10593) was purchased from Proteintech (USA). MZ1 (HY-107425) was purchased from MedChemExpress (USA). MG-132 (S2619) was purchased from Selleck (USA).

### Stable cell establishment for SDC1 knockdown

SDC1-specific shRNA and scrambled shRNA plasmids were constructed by GENEWIZ (China). Lentiviral particle generation was described previously [36]. In brief, HEK293T cells were cultured in a 10 cm dish until they reached 80% confluence. Subsequently, the cells were co-transfected with target plasmid (8 μg), psPAX2 (6 μg), and pMD2.G (2 μg), using PEI (Sigma, USA). After thorough washing and replenishment with a fresh medium, the cells were then incubated for an additional 30 h. Subsequently, the supernatant enriched with lentiviral particles was collected, filtered, and preserved by freezing it at -80 °C. U251 and U87 cells were subjected to infection with serial dilutions of lentiviral supernatant. The sequence for shVHL was 5’-CTCAACTTCGACGGCGAGC-3’, the sequence for shSDC1 was 5’-GACTGCTTTGGACCTAAAT-3’; the sequence for shNC was 5’-TTCTCCGAACGTGTCACGT-3’.

### RNA extraction and qRT-PCR

Total RNA was extracted using TRIzol reagent (Invitrogen, USA). Subsequently, cDNA was synthesized following the manufacturer’s protocol with HiScript III All-in-one RT SuperMix (Vazyme, China). Quantitative real-time reverse transcription PCR (qRT-PCR) was carried out using 2 × Taq Pro Universal SYBR qPCR Master Mix (Vazyme, China). The relative mRNA levels were calculated after normalization against GAPDH. The sequences of relative primers are provided below:

- BRD4, F: 5′-CGCTATGTCACCTCCTGTTTGC-3′ and R: 5′-ACTCTGAGGACGAGAAGCCCTT-3′.
- SDC1, F: 5′-TCCTGGACAGGAAAGAGGTGCT-3′ and R: 5′-TGTTTCGGCTCCTCCAAGGAGT-3′.
- CyclinB, F: 5′-TCGCCTGAGCCTATTTTGGT-3′ and R: 5′-GCATCTTACTTGGGCACACAA-3′.
- CDC2: F: 5′-AGTCTGGTCTTTCTTTGGCTGTCAG-3′ and R: 5′-AAACACCTACAACCACCACTCTGC-3′.
- GAPDH, F: 5′-ATCATCCCTGCCTCTACTGG-3′ and R: 5′-CCCTCCGACGCCTGCTTCAC-3′.

### Western blotting

To extract proteins, cells were harvested by centrifugation at 1000 rpm under 4 °C for 5 min. The cell pellets underwent two rounds of washing with PBS and were reconstituted in a suitable RIPA protein lysis buffer (Beyotime, China). The protein concentrations were assessed utilizing the BCA Protein Quantification Kit (Vazyme, China).

Western blotting was performed by loading protein samples onto SDS-PAGE gels, followed by electrophoretic transfer to PVDF membranes (Millipore, Germany). Blocking was carried out using 5% non-fat milk for 1 h at room temperature. Prior to incubation with the primary antibody, the membrane was cut into strips of suitable width, determined by the molecular weight of the target protein corresponding to the antibody. This procedure aimed to optimize the incubation with the primary antibody. The membranes were subsequently exposed to primary antibodies at 4 °C overnight. Next, the membranes were treated with secondary antibodies labeled with HRP for 1 h at room temperature, and result detection was carried out using the Super Signal West Dura Extended kit (Thermo Scientific, USA). GAPDH or β-actin served as the internal control.

### Cell proliferation and colony formation assay

Cell proliferation assessments were conducted using the CCK-8 assay method. Briefly, CCK-8 (DOJINDO, Japan) was added to each well, followed by incubation for 2 h. Following this incubation, the absorbance at 450 nm was measured using a Microplate Absorbance Reader (Bio-Rad, USA).

To conduct the colony formation assay, cells were seeded at a density of 1,000 cells per well in 6-well plates and exposed to the specified conditions for 14 days. After the incubation period, the cells were fixed with a 4% formaldehyde solution and then stained with 0.1% crystal violet dye for 1 h.

### EdU staining analysis

EdU labeling was carried out using the BeyoClick™ EdU Cell Proliferation Kit (Beyotime, China) following the manufacturer’s guidelines. In brief, cells were cultured with EdU for 2 h. Subsequently, they were fixed with 4% paraformaldehyde for 10 min and blocked with Immunol Staining Blocking Buffer (Beyotime, China) for an additional 1 h, and then treated with the Click Reaction Mixture for 30 min, followed by a 5-min incubation with DAPI.

### Flow cytometric analysis

Cell cycle and apoptosis analyses were performed using the Cell Cycle Assay Kit and Annexin V-Alexa Fluor 488/PI (Fcmacs, China), respectively, following the manufacturer’s instructions. Briefly, cells were collected by centrifugation at 1000 rpm. For apoptosis analysis, cells were stained with Annexin V and PI, and the fluorescence was measured using the GALLIOS flow cytometer (Beckman Coulter, USA). To perform cell cycle analysis, cells were fixed in 80% ethanol overnight and subsequently stained with PI and RNase before measurement using the GALLIOS flow cytometer.

### In vivo procedure for GBM xenograft preparation and MZ1 treatment in nude mice

For the GBM subcutaneous transplanted tumor model, 5 × 10^6^ U87 cells were inoculated into the left flank of nude mice. Subsequently, these mice were randomly divided into two groups, each consisting of 6 individuals (*n* = 6). Day 0 was designated as the day the tumor was established. Starting from the 3rd day post-tumor inoculation, the drug treatment group received intraperitoneal injections of MZ1 at a dose of 12.5 mg/kg every 2 days, following a previously established protocol [37]. The vehicle group received an equivalent dose of 5% Kolliphor^®^ HS15 as a substitute for MZ1. The mice’s body weight and tumor volume were monitored every three days. The survival endpoint was defined as the point at which the tumor in the vehicle group exceeded 1 cm^3^. Subsequently, all nude mice were humanely euthanized via intraperitoneal administration of 120 mg/kg ketamine and 150 mg/kg xylazine (2:l solution). The experimental procedures for all mice followed the Regulations for the Administration of Affairs Concerning Experimental Animals, as approved by the Animal Ethics Committee of Soochow University. ARRIVE guidelines (http://arriveguidelines.org) were followed.

### RNA‑sequencing (RNA‑seq) and data analysis

RNA-seq was conducted following the provided protocols by Novogene (China). We employed RNA-seq to analyze gene expression profiles in U87 cells exposed to either 400 nM MZ1 or an equivalent volume of DMSO for 48 h. Total RNA extraction was performed with TRIzol reagent. Novogene carried out the RNA purification, library construction, sequencing processes, and data analysis. The original RNA-seq data has been deposited in the Gene Expression Omnibus (GEO) database (the accession number GSE244878).

### Chromatin immunoprecipitation sequencing (ChIP-seq) data analysis

U87 cells were plated in T75 flasks and subsequently divided into two cohorts. The treatment group received exposure to 400 nM of MZ1 for a duration of 48 h, while the control group was subjected to an equivalent volume of solvent for the same period. The ChIP experiment was conducted following the established protocol as described previously [23]. In brief, a total of 3 × 10^7^ U87 cells were subjected to ChIP following the established protocol. Initially, the cells were cross-linked with 1% paraformaldehyde. Subsequently, the reaction was terminated by glycine for 5 min. After centrifugation, the cells were lysed in cell lysis buffer with a protease inhibitor. Following cell lysis, the cells were disrupted by gentle aspiration using a 1 ml insulin needle. The resulting precipitate was resuspended in a shearing buffer supplemented with a protease inhibitor. Subsequently, the chromatin was sonicated to yield DNA fragments ranging from 300 to 800 bp in length. The supernatant was subjected to immunoprecipitation with an H3K27Ac antibody (Abcam, USA) at 4 ℃ overnight. The following day, Dynabeads Protein G beads (Thermo Fisher Scientific, USA) were added to facilitate immunoprecipitation reactions at 4 ℃ for 4 h. The antibody-chromatin complexes attached to the beads were washed with lysis buffer and then washed with TE buffer. The antibody-chromatin complexes were eluted from the beads using elution buffer and treated with 5 M NaCl at 65 ℃ overnight. To remove any contaminating RNAs, RNase (CST, USA) was added at 37 ℃ for 30 min. Subsequently, Proteinase K (Invitrogen, USA), 1 M Tris HCl (pH 8.0), and 0.5 M EDTA (pH 8.0) were added and incubated at 45 ℃ for 1 h. The DNA fragments were subjected to purification using the PCR Purification Kit (QIAGEN, Germany). The original ChIP-seq data has been deposited in the Gene Expression Omnibus (GEO) database (the accession number GSE244893).

### Statistical analysis

We performed statistical analyses using GraphPad Prism 9.0.0 software (USA). Differences between the two groups were examined with a two-tailed paired *Student’s t*-test. Variations among multiple groups were assessed using a one-way analysis of variance (ANOVA). Statistical significance was defined as *p*-values below 0.05 (*p < 0.05, **p < 0.01). The results are expressed as means ± standard deviation (SD).

## Result

### Elevated BRD4 levels were linked to unfavorable outcomes in patients with GBM

**Fig. 1:**
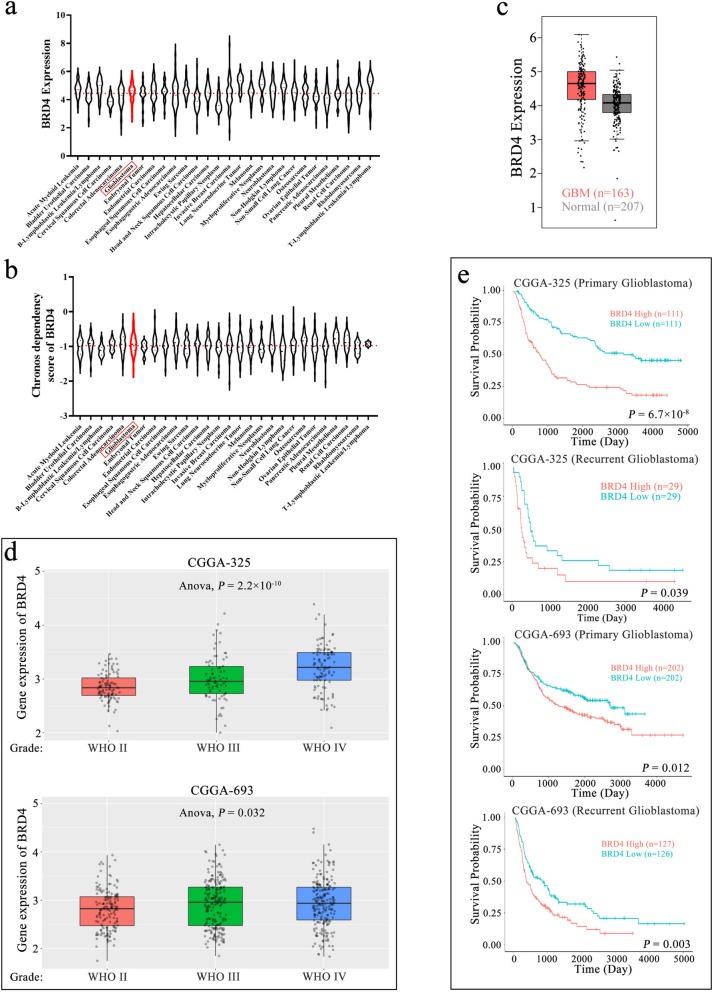
High expression of BRD4 was associated with poor prognosis and malignancy in GBM. **a**, **b** The expression of BRD4 and Chronos dependency score in various tumor cell lines. The BRD4 expression data and Chronos dependency score in tumor cell lines were sourced from the Cancer Cell Line Encyclopedia (CCLE, https://sites.broadinstitute.org/ccle). BRD4 expression levels and Chronos dependency score in GBM cell lines were marked in red. **c** The expression of BRD4 in clinical GBM patient tissues and normal tissues. Data were sourced from Gene Expression Profiling Interactive Analysis (GEPIA2, http://gepia2.cancer-pku.cn/#index). The red box represented GBM tissue (*n* = 207), while the gray box represented normal tissue (*n* = 163). **d** The expression of BRD4 in GBM patients with different degrees of malignancy. The data was derived from the Chinese Glioma Genome Atlas (CGGA, http://www.cgga.org.cn/index.jsp), where CGGA-325 represented data from the mRNAseq_325 dataset, and CGGA-693 represented data from the mRNAseq_693 dataset. Statistical analysis was performed using ANOVA. **e** Kaplan-Meier analysis examining the correlation between high or low BRD4 expression and the survival probability of patients with both primary and recurrent GBM. The data was derived from the Chinese Glioma Genome Atlas (CGGA, http://www.cgga.org.cn/index.jsp), where CGGA-325 represented data from the mRNAseq_325 dataset, and CGGA-693 represented data from the mRNAseq_693 dataset

To investigate the potential roles of BRD4 in tumors, we initially assessed the expression of BRD4 in diverse tumor cell lines through the CCLE database. The findings revealed that BRD4 was consistently upregulated in tumor cell lines, with its expression levels notably elevated in GBM compared to the overall average (Fig. 1a). The Chronos dependency score is derived from data obtained through a cell depletion assay. A lower Chronos score implies a greater probability that the gene under consideration is indispensable in a specific cell line. A score of 0 signifies that a gene is non-essential, while -1 is equivalent to the median score among all universally essential genes [38]. Through an examination of the CCLE database, we have ascertained that the average Chronos dependency score for BRD4 in diverse tumor cell lines hovers around -1. This underscores BRD4’s pervasive and substantial involvement in the biological processes of tumor cells, including GBM (Fig. 1b). In order to investigate the role of BRD4 in GBM, we initially examined the expression of BRD4 in GBM tissues and normal tissues using the GEPIA2 database. The findings indicated that the expression of BRD4 in GBM tissues was elevated compared to normal tissues (Fig. 1c). Moreover, upon delving deeper into the CGGA database, we uncovered a correlation between the escalating malignancy of GBM and an increase in BRD4 expression (Fig. 1d). Notably, elevated BRD4 expression was markedly linked to unfavorable prognoses in both primary and recurrent GBM patients (Fig. 1e). Collectively, these data indicated that the aberrant expression of BRD4 played a role in tumor initiation and was correlated with a less favorable prognosis in GBM patients. These results emphasized the potential importance of targeting BRD4 as a therapeutic option for individuals with GBM.

### MZ1 inhibited the proliferation of GBM cell lines

**Fig. 2:**
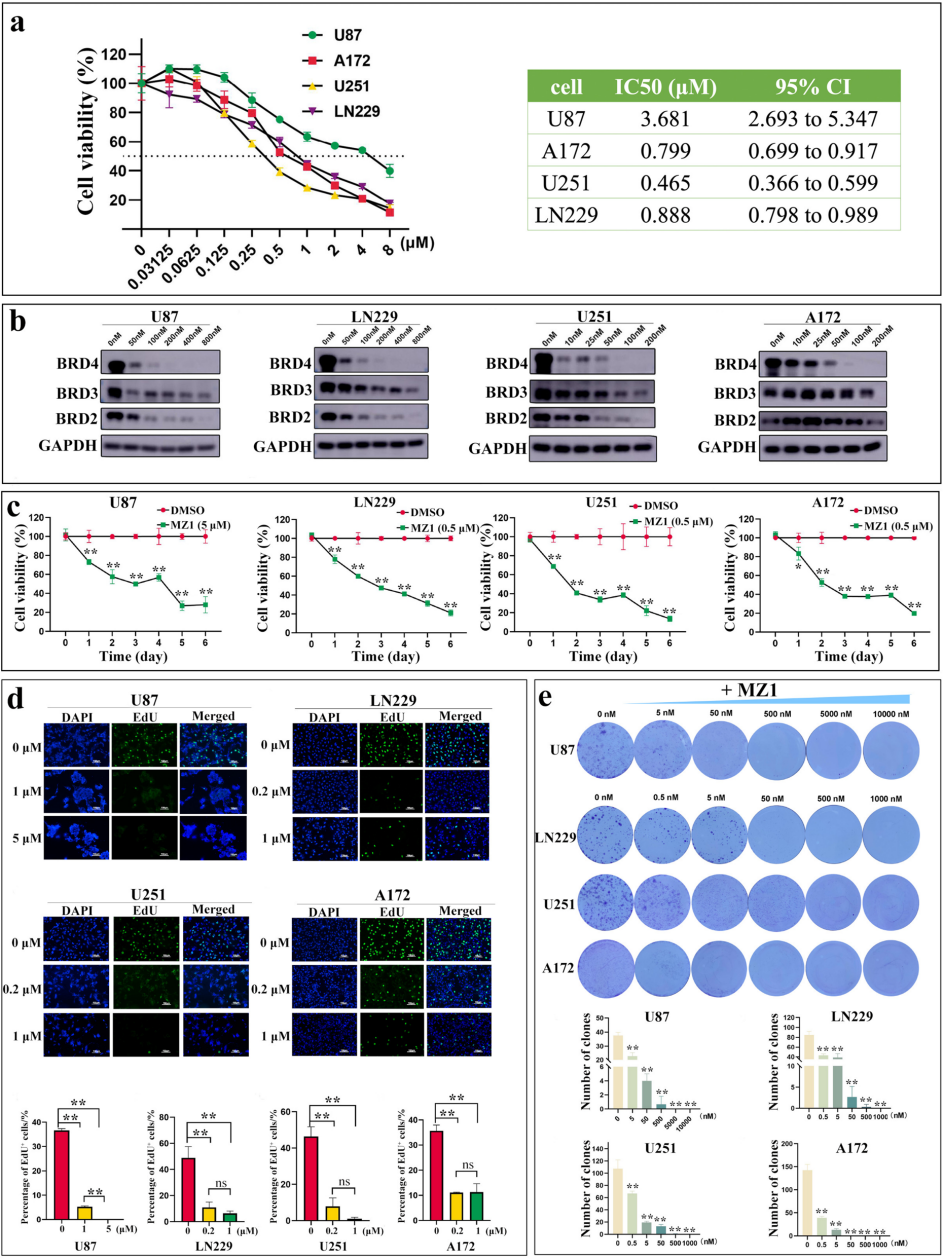
MZ1 dose-dependently inhibited BRD4 protein and GBM cell proliferation. **a** Left panel: Cell viability of GBM cells after treatment with MZ1 at the indicated concentrations for 72 h, 0 μM corresponded to a final concentration of 1% DMSO. Green circles represented U87, red squares represented A172, yellow triangles represented U251, and purple inverted triangles represented LN229. Right panel: The IC50 value of MZ1 in different GBM cell lines. **b** Western blot analysis was performed to assess the protein levels of BRD2, BRD3, and BRD4 after treatment with MZ1 at the indicated concentrations for 48 h, 0 nM corresponded to a final concentration of 1% DMSO, using GAPDH as an internal reference. **c** CCK-8 assay was used to measure the changes in cell viability at different time points after treatment with various concentrations of MZ1, as shown in the figure. Red circles represented 1% DMSO treatment, and green squares represented the concentration of MZ1 treatment as depicted in the figure. **d** EdU incorporation experiment was conducted to detect the number of EdU-positive cells after 72 h of treatment with the concentrations of MZ1 as shown in the figure, with 0 μM representing the final concentration of 1% DMSO treatment. **e** A clone formation experiment was conducted to measure the number of cell clones formed after 14 days of treatment with the concentrations of MZ1 as shown in the figure, with 0 nM representing the final concentration of 1% DMSO treatment. Data was presented as Mean ± SD. *Student’s t*-test, *p < 0.05, **p < 0.01; ns, non-significant

In the previous findings, we discovered a robust association between elevated BRD4 expression and unfavorable prognoses among GBM patients. To further investigate the role of BRD4 in GBM, we employed the BRD4 inhibitor MZ1 to target four GBM cell lines. The results revealed that MZ1 could effectively inhibit GBM cell lines at relatively low concentrations, with IC50 values of 3.68 μM for U87, 0.89 μM for LN229, 0.80 μM for A172, and 0.47 μM for U251 (Fig. 2a). To ascertain the selectivity of MZ1 towards BET family proteins, we treated GBM cells with varying concentrations of MZ1 and assessed the protein levels of BET family proteins, including BRD2, BRD3, and BRD4. The results revealed that MZ1 could inhibit BET family protein expression in a dose-dependent manner, with a higher inhibitory efficiency observed for BRD4 compared to other BET family proteins (Fig. 2b). To further elucidate the impact of MZ1 on the viability of GBM cells, we assessed changes in cell proliferation following MZ1 treatment using CCK-8, EdU incorporation, and colony formation experiments. The results revealed that MZ1 can dose-dependently inhibit the proliferation of GBM cells (Fig. 2c-e). The above results indicated that the BRD4 inhibitor MZ1 could significantly suppress the proliferation of GBM cells in vitro.

### MZ1 induced apoptosis and cell cycle arrest in GBM cells

**Fig. 3:**
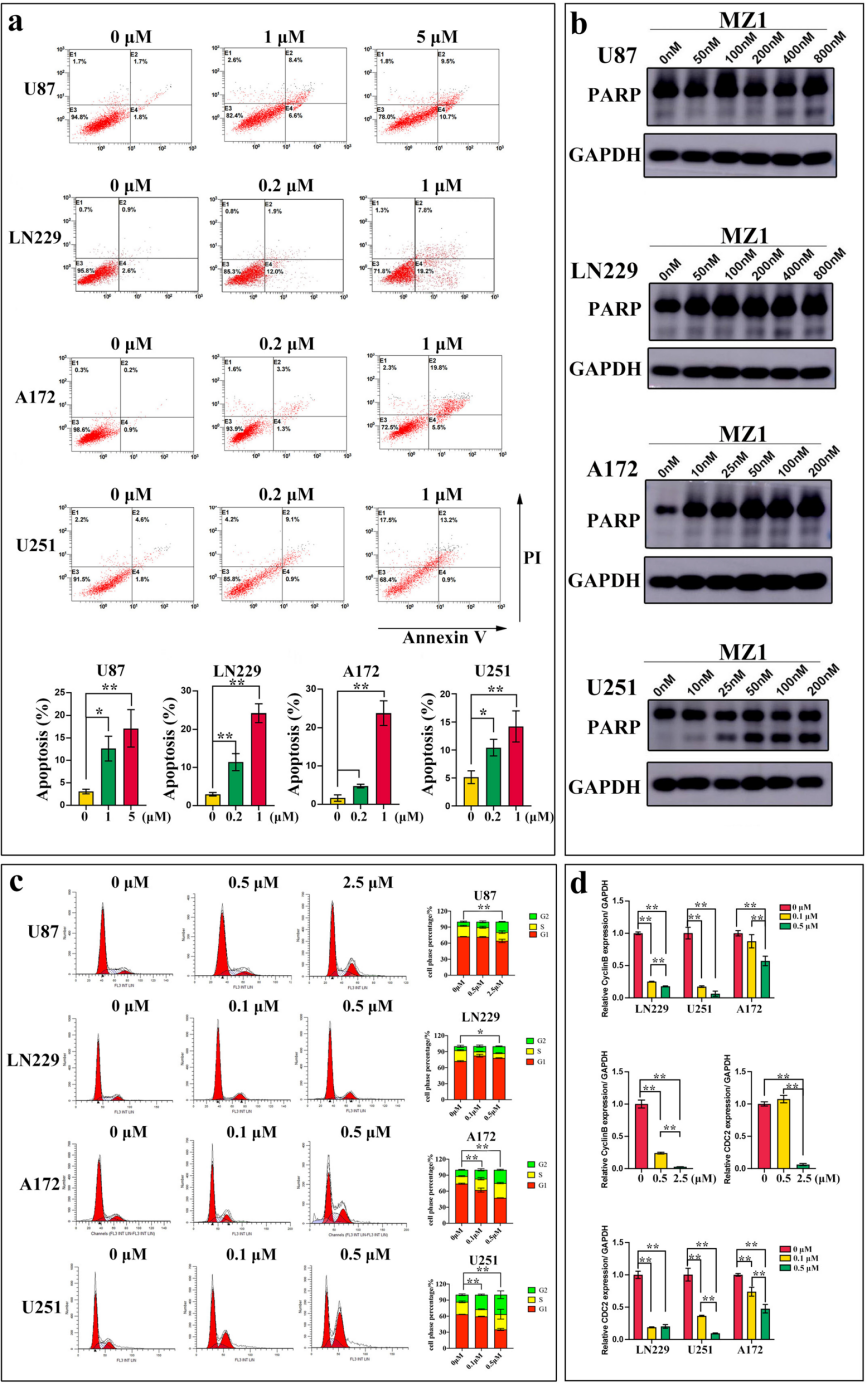
MZ1 induced the GBM cell cycle G2 arrest and apoptosis. **a** The apoptosis ratio of cells after treatment with the concentrations of MZ1 as shown in the figure for 72 h, with 0 μM representing the final concentration of 1% DMSO treatment. **b** Western blot analysis was performed to assess the protein cleavage status of PARP after treatment with the concentrations of MZ1 as shown in the figure for 72 h, with 0 nM representing the final concentration of 1% DMSO treatment, using GAPDH as an internal reference. **c** The proportions of cell cycle phases after treatment with the concentrations of MZ1 as shown in the figure for 72 h, with 0 μM representing the final concentration of 1% DMSO treatment. **d** Real-time fluorescence quantitative PCR was used to measure the mRNA levels of Cyclin B and CDC2 after treatment with the concentrations of MZ1 as shown in the figure for 72 h, with 0 μM representing the final concentration of 1% DMSO treatment, using GAPDH as an internal reference. Data was presented as Mean ± SD. *Student’s t*-test, *p < 0.05, **p < 0.01; ns, non-significant

Previous studies have found that inhibiting BRD4 can induce cell cycle arrest and trigger apoptosis in tumor cells [39]. Consequently, we conducted further investigations to assess the effects of MZ1 on the cell cycle and apoptosis of GBM cells. The results revealed that MZ1 could dose-dependently induce apoptosis in GBM cells (Fig. 3a). The cleavage of PARP is one of the markers of cell apoptosis [40]. We observed that following MZ1 treatment, as the proportion of apoptotic cells increased, there was a corresponding elevation in the proportion of cleaved PARP in GBM cells (Fig. 3b). Furthermore, in comparison to the control group, MZ1 treatment significantly induced cell cycle G2 phase arrest in GBM cells, and with increasing concentrations of MZ1, the proportion of cells in the G2 phase also correspondingly increased (Fig. 3c). Cyclin B1 and CDC2 are key genes involved in the transition from the G2 phase to the M phase of the cell cycle [41]. In this regard, we detected, through RT-qPCR, that MZ1 treatment, in comparison to the control group, could concentration-dependently downregulate the mRNA levels of Cyclin B1 and CDC2 (Fig. 3d). The above results indicated that MZ1 could exert its inhibitory effect on GBM cell proliferation by inducing cell cycle G2 phase arrest and apoptosis.

### The VHL protein served as a crucial mediator for the function of MZ1

**Fig. 4:**
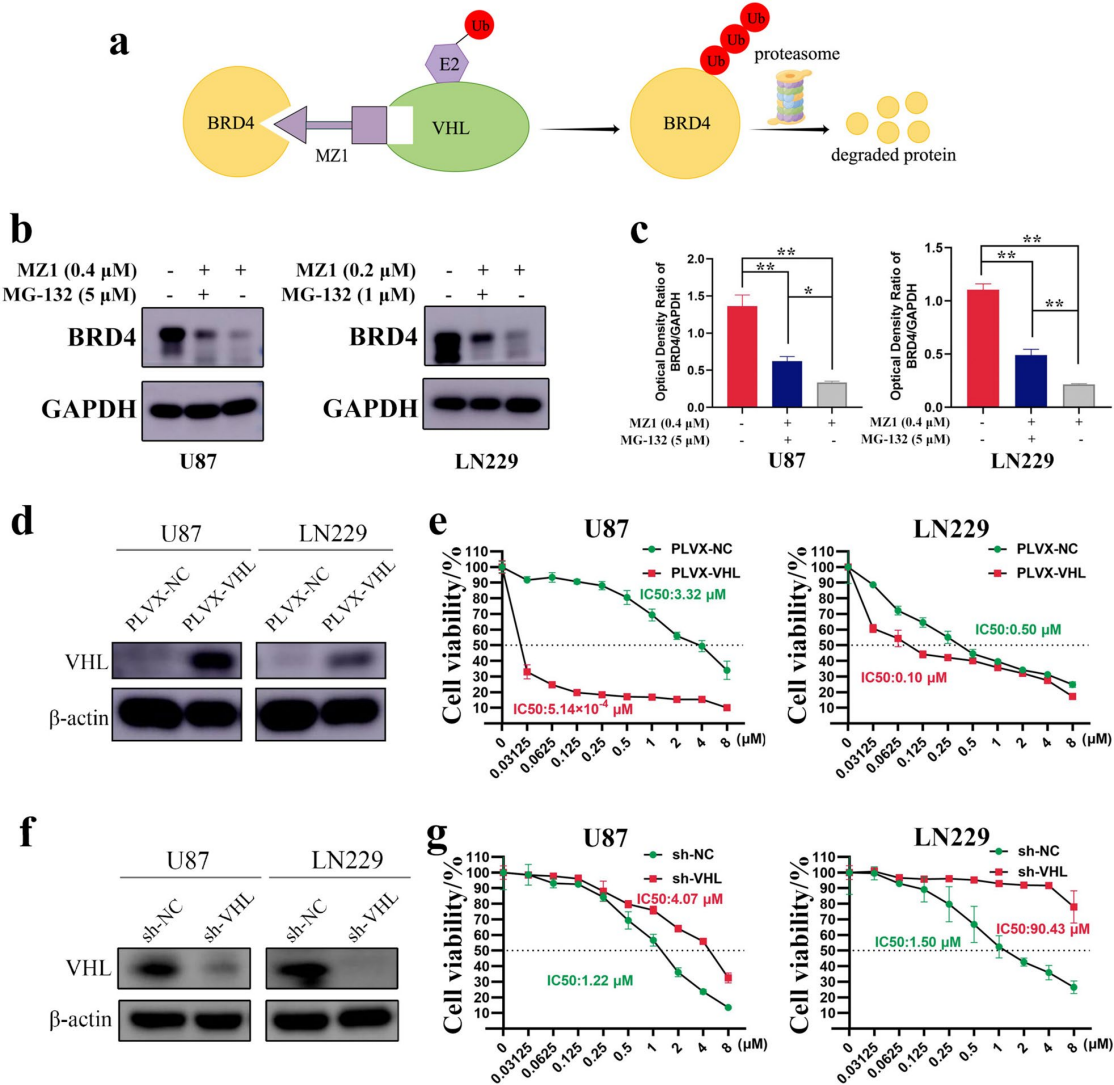
VHL mediated the degradation of BRD4 through the ubiquitin-proteasome pathway. **a** Mechanism of action diagram for MZ1. **b** U87 and LN229 cells were treated with MZ1 at the concentrations indicated in the figure for 48 h (- represented 1% DMSO, + represented MZ1). Subsequently, cells were further treated with MG-132 at the concentrations shown in the figure for 8 h (- represents 1% DMSO). Western blot analysis was performed to measure the protein levels of BRD4, with GAPDH serving as an internal reference. **c** Based on the grayscale values of the bands in (**B**), the ratio of BRD4 protein to the corresponding GAPDH protein was calculated for each group. **d** Western blot was performed to assess the overexpression of VHL in U87 and LN229 cells. PLVX-NC represented the empty vector group, PLVX-VHL represented the VHL overexpression group, and β-actin was used as an internal reference. **e** Treatment of U87 and LN229 cells overexpressing empty vector or VHL with the concentrations of MZ1 as shown in the figure for 72 h. Cell viability compared to 0 μM (1% DMSO) was assessed using CCK-8. Green dots represented the empty vector group, and red squares represented the VHL overexpression group. **f** Western blot was performed to assess the knockdown of VHL in U87 and LN229 cells. sh-NC represented the negative control group, sh-VHL represented the VHL knockdown group, and β-actin was used as an internal reference. **g** U87 and LN229 cells with negative control or VHL knockdown were treated with the concentrations of MZ1 as shown in the figure for 72 h. Cell viability compared to 0 μM (1% DMSO) was assessed using CCK-8. Green dots represented the negative control group, and red squares represented the VHL knockdown group. Data was presented as Mean ± SD. *Student’s t*-test, *p < 0.05, **p < 0.01; ns, non-significant

As mentioned earlier, MZ1 achieves the inhibition of BRD4 by linking it to the E3 ubiquitin ligase VHL, thereby facilitating the ubiquitin–proteasome system (UPS) degradation of BRD4 (Fig. 4a). To validate whether the reduction of BRD4 protein induced by MZ1 was mediated via the UPS pathway, we co-administered the proteasome inhibitor MG-132 during MZ1 treatment of GBM cells. Western blot experiments demonstrated that MZ1 treatment substantially reduced BRD4 protein levels, and MG-132 partially reversed the MZ1-induced downregulation of BRD4 protein, confirming the mechanism of function of MZ1 (Fig. 4b, c). To further validate the critical role of VHL in mediating the effects of MZ1, VHL was overexpressed or knocked down in GBM cells. Cell viability assays revealed that, in comparison to the control group, the overexpression of VHL led to a notable reduction in the IC50 of MZ1 (Fig. 4d, e), whereas VHL knockdown substantially increased the IC50 of MZ1 (Fig. 4f, g). Taken together, these results strongly indicated that VHL plays a crucial role in enabling MZ1 to exert its anti-tumor effect in GBM cells.

### MZ1 inhibited the growth of GBM cells in vivo

**Fig. 5:**
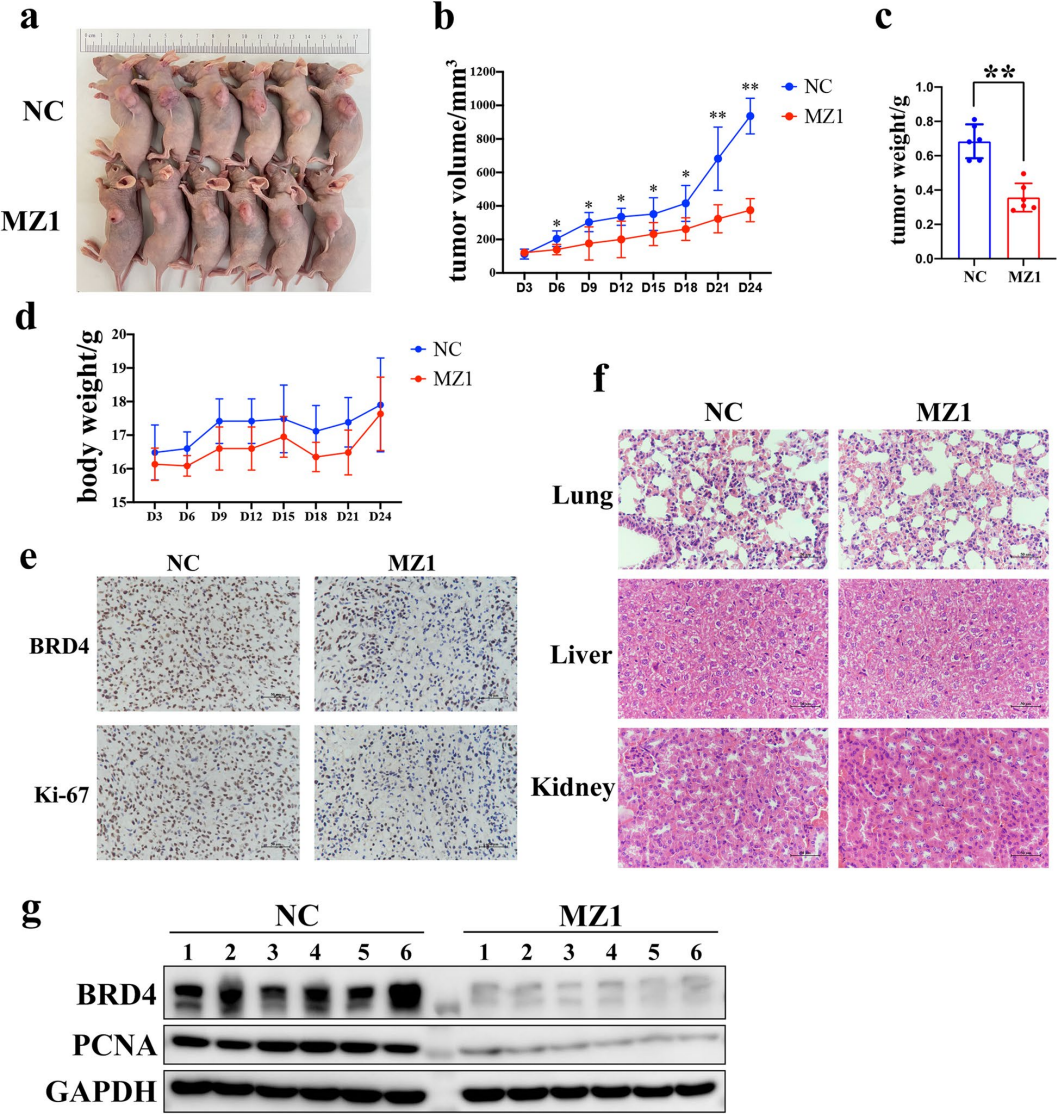
MZ1 inhibited the development of transplanted tumors in nude mice. **a** The growth of transplanted tumors under the armpits of nude mice in the control group (NC) and the MZ1-treated group. **b** Tumor volume statistics on the days as shown in the figure for the control group (NC) and the MZ1-treated group. The blue line represented the control group, while the red line represented the MZ1-treated group. **c** Final tumor mass statistics for the control group and the MZ1-treated group. **d** Weight statistics for mice in the control group (NC) and the MZ1-treated group on the days as shown in the figure. The blue line represented the control group, while the red line represented the MZ1-treated group. **e** IHC staining was performed to assess the protein expression of BRD4 and Ki-67 in tumors from the control group (NC) and the MZ1-treated group. The black scale bar represented 50 μm. **f** HE staining was performed to assess the tissue damage in the lungs, liver, and kidney of mice from the control group (NC) and the MZ1-treated group. The black scale bar represented 50 μm. **g** Western blot was used to measure the protein expression levels of BRD4 and PCNA in tumors from the control group (NC) and the MZ1-treated group, with GAPDH serving as the internal reference. Data was presented as Mean ± SD. *Student’s t*-test, *p < 0.05, **p < 0.01; ns, non-significant

To further assess the feasibility and effectiveness of MZ1 in treating GBM in vivo, the GBM model was established by subcutaneously xeno-transplanting U87 cells into nude mice. The results revealed that, in comparison to the control group, MZ1 treatment significantly inhibited the development of GBM cells in vivo and had no significant impact on the change in mouse body weight (Fig. 5a-d). Furthermore, our observations through IHC staining indicated that MZ1 treatment significantly inhibited the expression of BRD4 and the proliferation marker Ki-67 in tumor tissues, in comparison to the control group (Fig. 5e). To evaluate the toxicity of MZ1 in mice, we conducted HE staining and determined that, in comparison to the control group, MZ1 treatment did not result in significant damage to the lungs, liver, and kidneys of the mice (Fig. 5f). Moreover, to further confirm the influence of MZ1 on BRD4 expression in tumor tissues in vivo, our analysis using RT-qPCR and Western blot showed that, in contrast to the control group, MZ1 treatment significantly decreased the protein levels of BRD4 and also lowered the protein levels of the proliferation marker PCNA in tumor tissues (Fig. 5g). The above results indicated that MZ1 could significantly inhibit the occurrence and development of GBM in vivo.

### RNA-seq and ChIP-seq combined analysis to identify downstream target genes

**Fig. 6:**
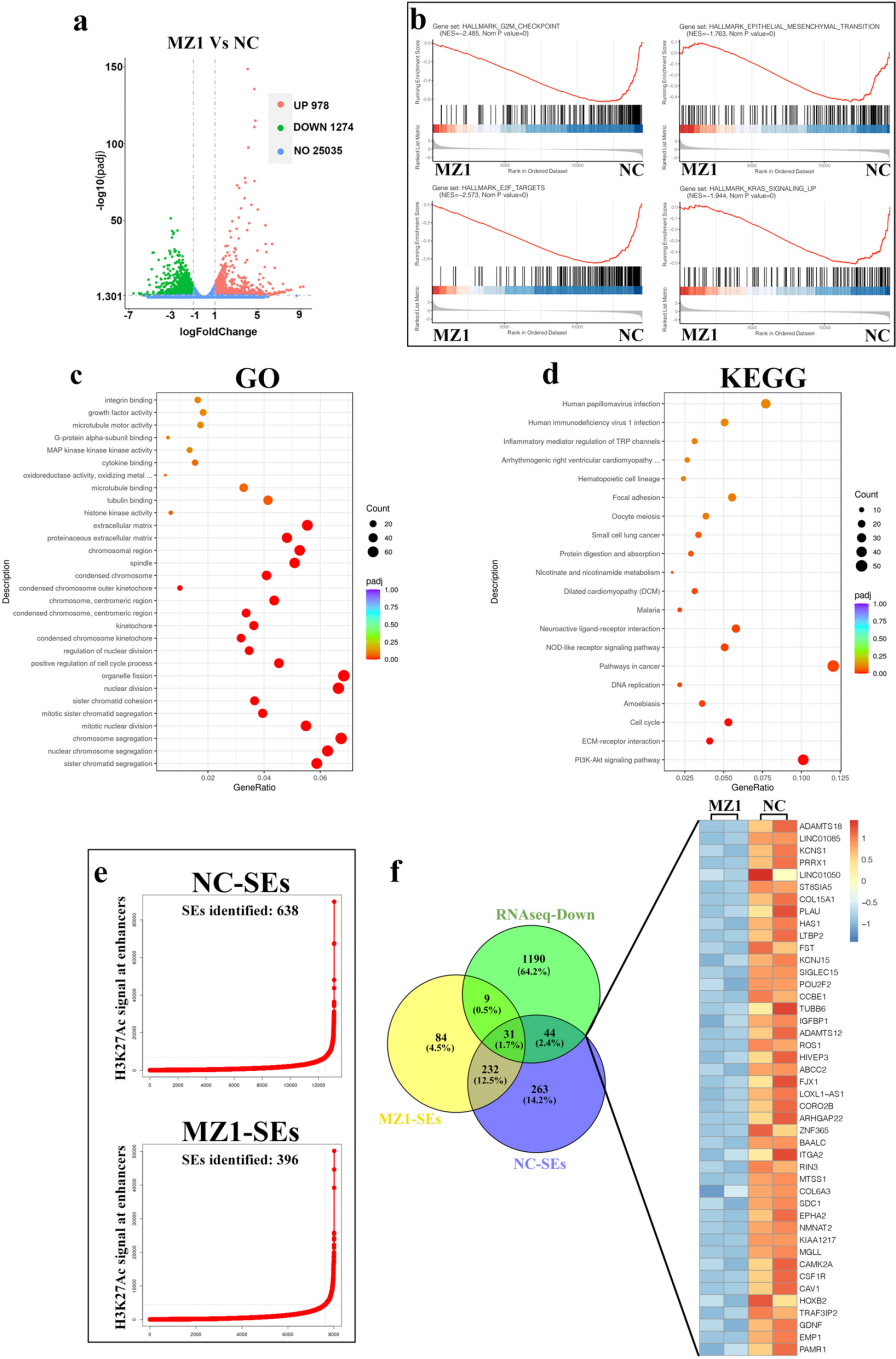
RNA-seq combined with ChIP-seq for the discovery of novel candidate genes. **a** Volcano plot of differential gene expression in U87 cells treated with NC (1% DMSO) and MZ1. The green and red dots represented all statistically significant genes with downregulated or upregulated expression (log2FoldChange < -1.0 or > 1, adjusted *p*-value < 0.05). **b** Enrichment analysis results of differentially expressed genes were performed using the GSEA Pathway Database to elucidate the functional role of MZ1 in gene regulation. **c**, **d** Using GO and KEGG pathway databases to analyze the cellular processes and signaling pathways involved in the downregulated genes after MZ1 treatment. **e** Enhancers were ranked based on the ascending H3K27Ac signal in U87 cells treated with NC (1% DMSO) and MZ1. The number of SE-regulated genes and the cutoff value of each group were shown in the figure. **f** The Venn diagram illustrated the intersection of downregulated genes in RNA-seq, genes regulated by SEs in the NC group, and genes unaffected by SEs regulation after MZ1 treatment. The right panel depicted a heatmap of the expression levels of these genes in the NC and MZ1 treatment groups

Based on the experimental results mentioned above, it was clear that MZ1 demonstrated a substantial inhibitory impact on the proliferation of GBM, both in vitro and in vivo. To further investigate the potential mechanisms underlying the effects of MZ1, we conducted RNA-seq analysis to compare differences in gene expression between the control group and the MZ1 treatment group. The results revealed that, in comparison to the control group, the MZ1 treatment group showed the upregulation of 978 genes and the downregulation of 1274 genes (Fig. 6a). To clarify the function of MZ1 in gene regulation, we performed annotation and enrichment analysis on the functions of the downregulated genes using HALLMARK pathway analysis. The findings revealed that the genes influenced by MZ1 were linked to the regulation of multiple tumor-related processes and activities, including the G2M checkpoint, epithelial-mesenchymal transition, E2F targets, and KRAS signaling (Fig. 6b). Furthermore, through GO and KEGG analysis, we discovered that the genes downregulated after MZ1 treatment are extensively involved in cell mitosis, cell cycle processes, and the PI3K-AKT signaling pathway, providing initial insights into the anti-tumor mechanisms of MZ1 (Fig. 6c, d).

There is a hypothesis that MZ1 possesses the potential to target the transcriptional regulation of BRD4, disrupt the structure of super-enhancers (SEs), and impede the transcriptional activation of SE-dependent oncogenes. Thus, to assess the impact of MZ1 on genes associated with super-enhancers in GBM, we carried out ChIP-seq using the H3K27Ac antibody to detect alterations in SE-associated genes. The results indicated that there were 638 genes under the regulation of SEs in normal GBM cells, and following MZ1 treatment, the count of genes subjected to SE regulation decreased to 396 (Fig. 6e). In order to refine the list of candidates, we implemented a multi-criteria feature selection process for potential oncogenes in GBM, considering: (i) genes regulated by SEs in the control group, (ii) genes unaffected by SE regulation after MZ1 treatment, and (iii) genes that showed downregulation following MZ1 treatment according to RNA-seq results. Using this criterion, we have indeed identified a total of 44 candidate genes through our screening process (Fig. 6f).

### The discovery of SDC1 as a crucial target gene through which MZ1 exerted its function

**Fig. 7:**
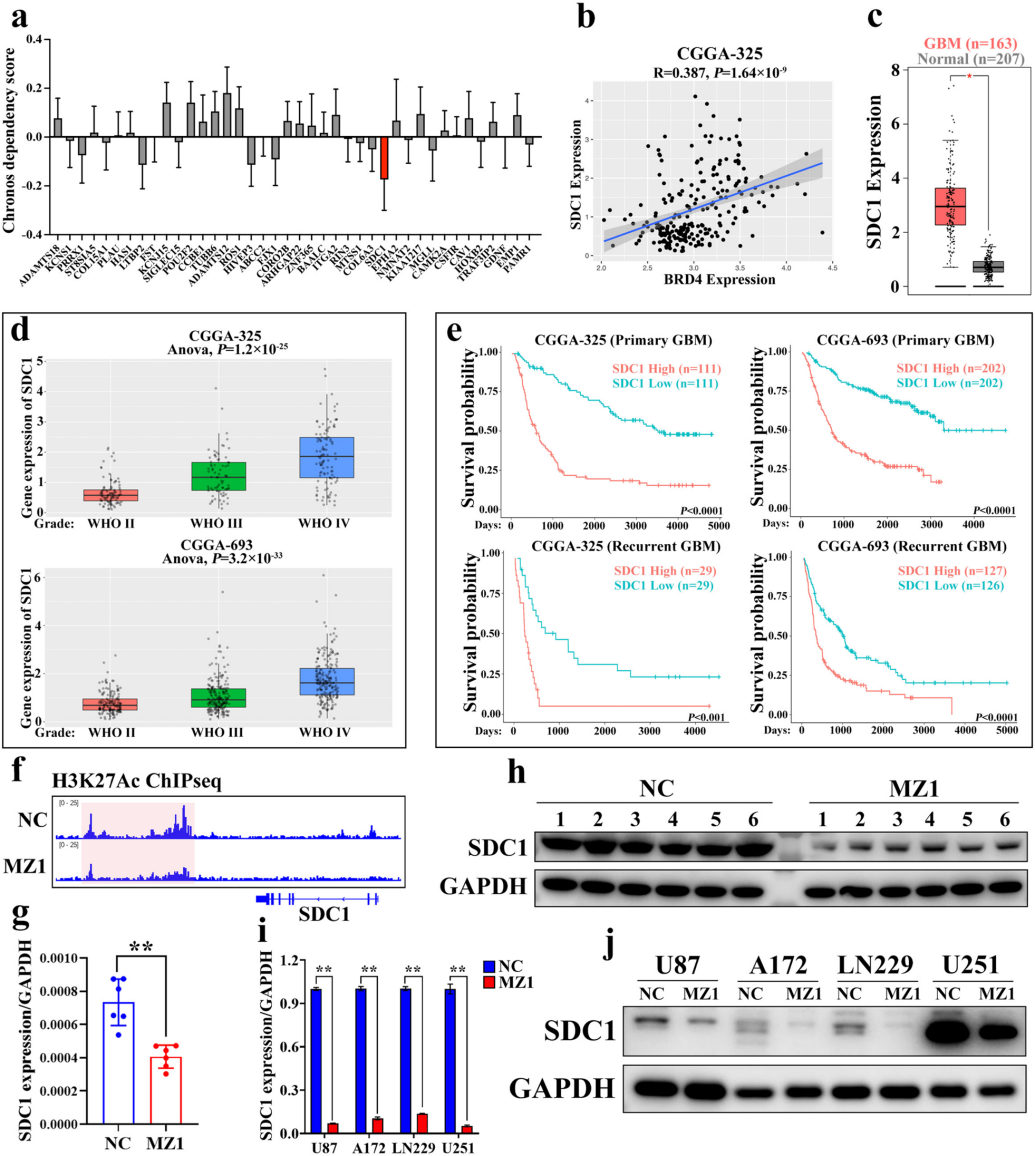
SDC1 was a potential oncogene in GBM regulated by BRD4. **a** The Chronos dependency scores for the previously selected genes in GBM cell lines from the CCLE database. The red bar graph represents the chronos dependency score for SDC1. **b** The correlation between SDC1 and BRD4 expression in GBM patient tissues. Data sourced from CGGA database (mRNAseq_325 dataset). **c** The expression of SDC1 in clinical GBM patient tissues and normal tissues. Data were sourced from the GEPIA2 database. The red box represented GBM tissue (*n* = 207), while the gray box represented normal tissue (*n* = 163). **d** The expression of SDC1 in GBM patients with different degrees of malignancy. The data was derived from the CGGA database, where CGGA-325 represented data from the mRNAseq_325 dataset, and CGGA-693 represented data from the mRNAseq_693 dataset. Statistical analysis was performed using ANOVA. **e** Kaplan-Meier analysis examining the correlation between high or low SDC1 expression and the survival probability of patients with both primary and recurrent GBM. The data was derived from the CGGA database, where CGGA-325 represented data from the mRNAseq_325 dataset, and CGGA-693 represented data from the mRNAseq_693 dataset. **f** Visualization of H3K27Ac modifications near the SDC1 gene in the genome of U87 cells treated with NC (1% DMSO) and MZ1 using IGV software. **g**, **h** The mRNA and protein expression levels of SDC1 in tumors from the control group (NC) and the MZ1-treated group, with GAPDH serving as the internal reference. **i**, **j** U87, A172, LN229, and U251 cells were treated with NC (1% DMSO) and MZ1 (1 μM for U87, and 0.2 μM for A172, LN229, and U251) for 72 h. The mRNA and protein levels of SDC1 in the cells were detected using RT-qPCR and Western blot, with GAPDH as an internal reference. Data was presented as Mean ± SD. *Student’s t*-test, *p < 0.05, **p < 0.01; ns, non-significant

As mentioned above, we screened 44 candidate target genes of MZ1. To further narrow down the selection, we compared the Chronos scores of these genes in GBM using the CCLE database (Fig. 7a). The findings highlighted that SDC1 exhibited the lowest Chronos score, implying the strongest association between SDC1 and the development of GBM. To explore the clinical correlation between SDC1 and BRD4 expression in GBM patients, we identified a robust positive correlation between the expressions of SDC1 and BRD4 using the CGGA database (Fig. 7b). Additionally, our observations using the GEPIA and CGGA databases revealed a markedly higher expression of SDC1 in GBM tissues when compared to normal tissues. Furthermore, with the escalating malignancy of GBM, there was a concomitant elevation in the expression of SDC1, and the heightened expression of SDC1 was strongly linked to an unfavorable prognosis in GBM (Fig. 7c-e). These findings further indicated the pivotal role of SDC1 in the development of GBM. To further validate the impact of MZ1 on SDC1 expression in GBM, we utilized IGV visualization of ChIP-seq results. Our observations revealed that, in comparison to the control group, there was a significant decrease in H3K27Ac modification levels within the enhancer region of the SDC1 gene following MZ1 treatment (Fig. 7f). Furthermore, both the mRNA and protein levels of SDC1 showed substantial downregulation in GBM subcutaneous xenografts and cell lines after MZ1 treatment (Fig. 7g-j). These findings collectively indicated that SDC1 was suppressed by MZ1 and exhibited a strong correlation with the occurrence and development of GBM in clinical contexts.

### Downregulating the expression of SDC1 inhibited the proliferation of GBM cells

**Fig. 8:**
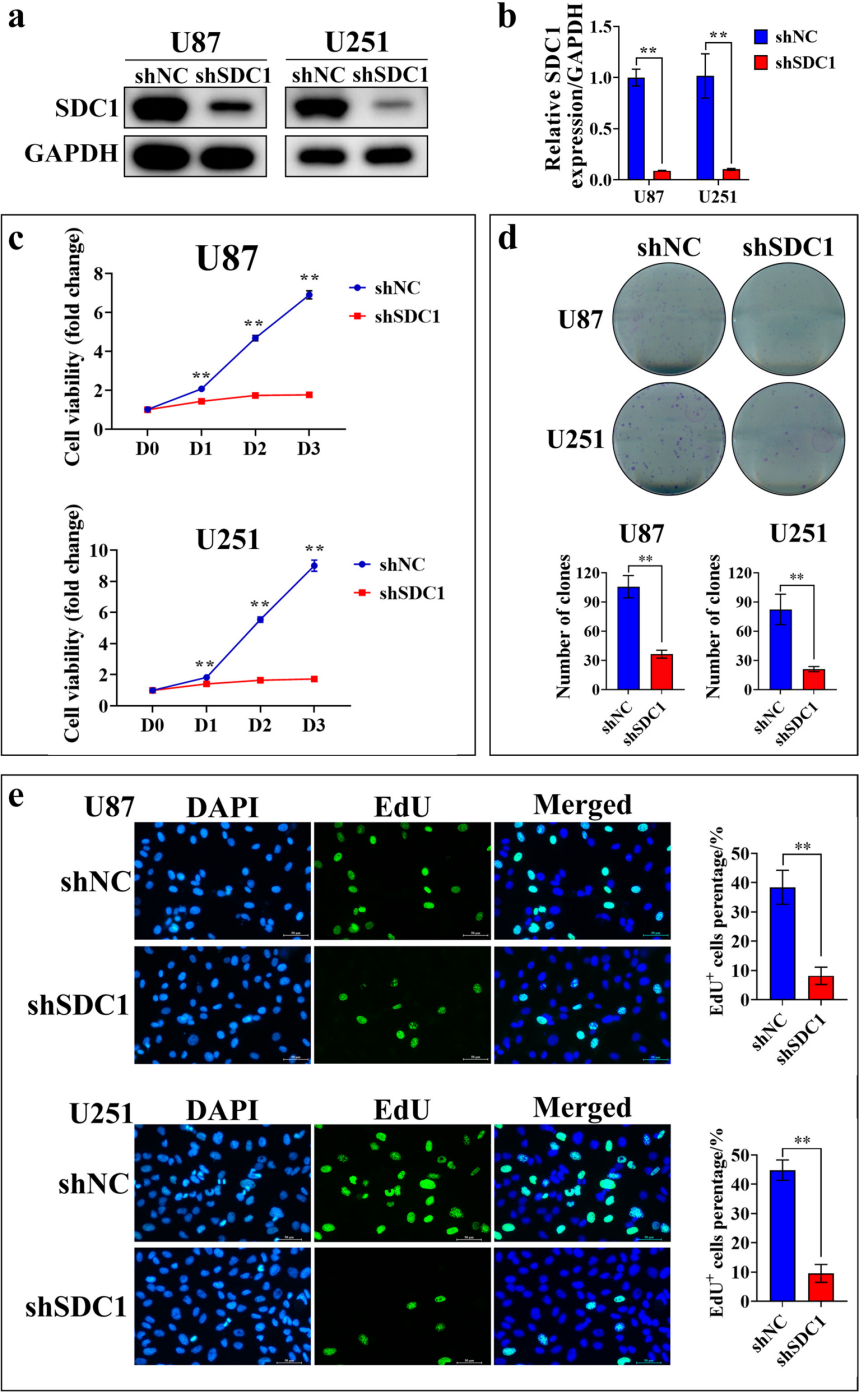
SDC1 was essential for GBM proliferation. **a**, **b** Western blot and RT-qPCR were performed to assess the knockdown of SDC1 in U87 and U251 cells. ShNC represented the negative control group, shSDC1 represented the SDC1 knockdown group, and GAPDH was used as an internal reference. **c** CCK-8 assay was performed to assess cell viability in U87 and U251 cells at the indicated time points. The control group (shNC, blue line) and SDC1 knockdown group (shSDC1, red line) were compared for cell activity, as shown in the figure. **d** A clone formation experiment was conducted to measure the number of cell clones formed after 14 days in the control group (shNC) and SDC1 knockdown group (shSDC1) of U87 and U251 cells. The bar chart represented a statistical summary of cell colony numbers. **e** EdU incorporation experiment was conducted to detect the number of EdU-positive cells in the control group (shNC) and SDC1 knockdown group (shSDC1) of U87 and U251 cells. The bar chart was a statistical representation of the proportion of EdU-positive cells. Data was presented as Mean ± SD. *Student’s t*-test, *p < 0.05, **p < 0.01; ns, non-significant

To elucidate the crucial role of SDC1 in GBM, we employed shRNA to reduce SDC1 expression in GBM cells (Fig. 8a, b). Our findings revealed that, when compared to the control group, the downregulation of SDC1 expression significantly suppressed the proliferation of GBM cells (Fig. 8c, d). Furthermore, EdU incorporation experiments exhibited that downregulating SDC1 expression significantly inhibited DNA replication in GBM cells (Fig. 8e). Taken together, the above results indicated that SDC1 played a crucial role in the normal growth of GBM cells.

## Discussion

GBM is among the most common primary brain tumors, known for its high malignancy, a median survival period of merely 9–12 months, and a five-year survival rate that does not exceed 5% [42]. Even after surgical resection, a significant recurrence risk persists [43]. Consequently, there is an immediate imperative to seek out more efficacious approaches for the clinical therapy of GBM. In recent years, research has revealed the widespread occurrence of epigenetic abnormalities in GBM [44]. In contrast to genetic mutations, epigenetics is reversible and can govern the transition between oncogenic and non-oncogenic states in GBM cells. BRD4, a reader of histone acetylation, serves as a therapeutic target in GBM, providing a novel research perspective on restoring the normal epigenetic profile of GBM cells and enhancing the treatment of malignant GBM [45].

Presently, numerous BRD4 inhibitors have been identified for their capacity to exert inhibitory effects on GBM through diverse pathways, both in vitro and in vivo, including JQ1 [46], OTX015 (MK-8628) [27], GEN987 [23]. Moreover, while no BET inhibitors have been granted FDA approval as of now, there are several drugs currently undergoing clinical trials at different stages for the treatment of various cancer types. These include CPI-0610 (NCT02157636) for multiple myeloma, RO6870810 (NCT02308761) for AML, and MK-8628 for TNBC, NSCLC, PDAC (NCT02259114), AML (NCT02698189), and GBM (NCT02296476), highlighting their substantial therapeutic potential. Nevertheless, at present, only MK-8628 has advanced to early-stage clinical trials for GBM treatment, underscoring the need for further research to comprehensively assess the therapeutic efficacy of BET inhibitors in GBM.

MZ1 is a novel inhibitor developed using PROTAC technology, which achieves its inhibition of BRD4 by promoting BRD4 ubiquitination and targeting it for proteasomal degradation. MZ1 has demonstrated significant anti-tumor effects in various types of cancers, including B-ALL [47], AML [48], NB [49], and breast cancer [50–52]. In this study, we utilized clinical databases of GBM patients to unveil a strong correlation between elevated BRD4 expression and aggressiveness as well as the poor prognosis of GBM. Furthermore, our in vitro and in vivo experiments demonstrated that MZ1 exerted its anti-GBM effects by inducing cell cycle arrest and apoptosis. RNA-seq analysis highlighted that genes downregulated after MZ1 treatment were extensively involved in processes like DNA replication, cell cycle progression, and Epithelial-Mesenchymal Transition. Additionally, ChIP-seq analysis unequivocally confirmed a marked reduction in genes regulated by SEs within GBM cells post MZ1 treatment. Collectively, these findings strongly suggested that MZ1 accomplished its anti-GBM effects by inhibiting BRD4 and disrupting SE structures. However, in order to advance the clinical translation of MZ1 for GBM treatment further, it is imperative to design experiments aimed at elucidating the pharmacokinetics, biodistribution, and metabolism of MZ1.

Additionally, SDC1 was identified as the most promising candidate gene through RNA-seq in combination with ChIP-seq after MZ1 treatment. SDC1 is a member of the heparan sulfate proteoglycan family and plays a crucial role in maintaining the typical cellular structure. It interacts with a variety of intracellular and extracellular proteins and facilitates signal transduction in response to environmental signals. The significant contribution of SDC1 to the promotion of tumorigenesis and metastasis is increasingly acknowledged across various cancer types, including GBM [53], PDAC [54], Multiple myeloma [55], Breast cancer [56], hepatocellular carcinoma [57], and Hodgkin’s lymphoma [58], suggesting the exciting potential of SDC1 as an innovative target for cancer treatment. Through our experiments, we have confirmed a close correlation between SDC1 and the malignancy level as well as poor prognosis of GBM. Significantly, the suppression of SDC1 has a pronounced inhibitory effect on the proliferation of GBM cells, further underscoring the potential of SDC1 as a therapeutic target for GBM.

Being a cell surface protein, SDC1 presents itself as a readily reachable candidate for drug development. Given its modified expression and pivotal function in promoting cancer advancement, SDC1 becomes an appealing prospect for cancer treatment, with various therapeutic approaches devised to address SDC1 in human cancers, such as BT062-DM4 [59], VIS832 [60], OC-46F2 [61] for Multiple myeloma, and Synstatin for breast cancer [62] and hepatocellular carcinoma [63]. Hence, further research is required to validate the clinical potential of SDC1 as a therapeutic target for GBM and to uncover the detailed molecular mechanisms through which SDC1 influences GBM and its interactions with other signaling pathways.

## Conclusion

In conclusion, our study demonstrated that MZ1 showed significant anti-tumor effects both in vitro and in vivo by selectively inhibiting BRD4 and SE-regulated oncogenes. Furthermore, we utilized a combination of RNA-seq and ChIP-seq to pinpoint exceptionally activated and compelling oncogenes in GBM cells. Our research has provided innovative therapeutic strategies and novel targets for the clinical treatment of GBM.

### Supplementary Information

**Supplementary material 1.**

## Data Availability

The data and material used to support the findings of this study are available from the corresponding author upon request. The original RNA-seq data has been deposited in the Gene Expression Omnibus (GEO) database (the accession number GSE244878). The original ChIP-seq data has been deposited in the Gene Expression Omnibus (GEO) database (the accession number GSE244893).

## Abbreviations

GBM: Glioblastoma Multiforme
BRD4: Bromodomain 4
SE: Super-enhancer
CGGA: Chinese Glioma Genome Atlas
GEPIA2: Gene Expression Profiling Interactive Analysis 2
CCLE: Cancer Cell Line Encyclopedia
BET: Bromodomain and extra-terminal
PROTAC: Proteolytic targeting chimera
EMT: Epithelial-mesenchymal transition
ChIP: Chromatin immunoprecipitation
GEO: Gene expression omnibus
