# Supplementary figures and images for: MZ1, a BRD4 inhibitor, exerted its anti-cancer effects by suppressing SDC1 in glioblastoma
Source: BMC Cancer. 2024 Feb 16;24:220. doi: 10.1186/s12885-024-11966-8 (PMC10870565; doi:10.1186/s12885-024-11966-8)

figure 2b

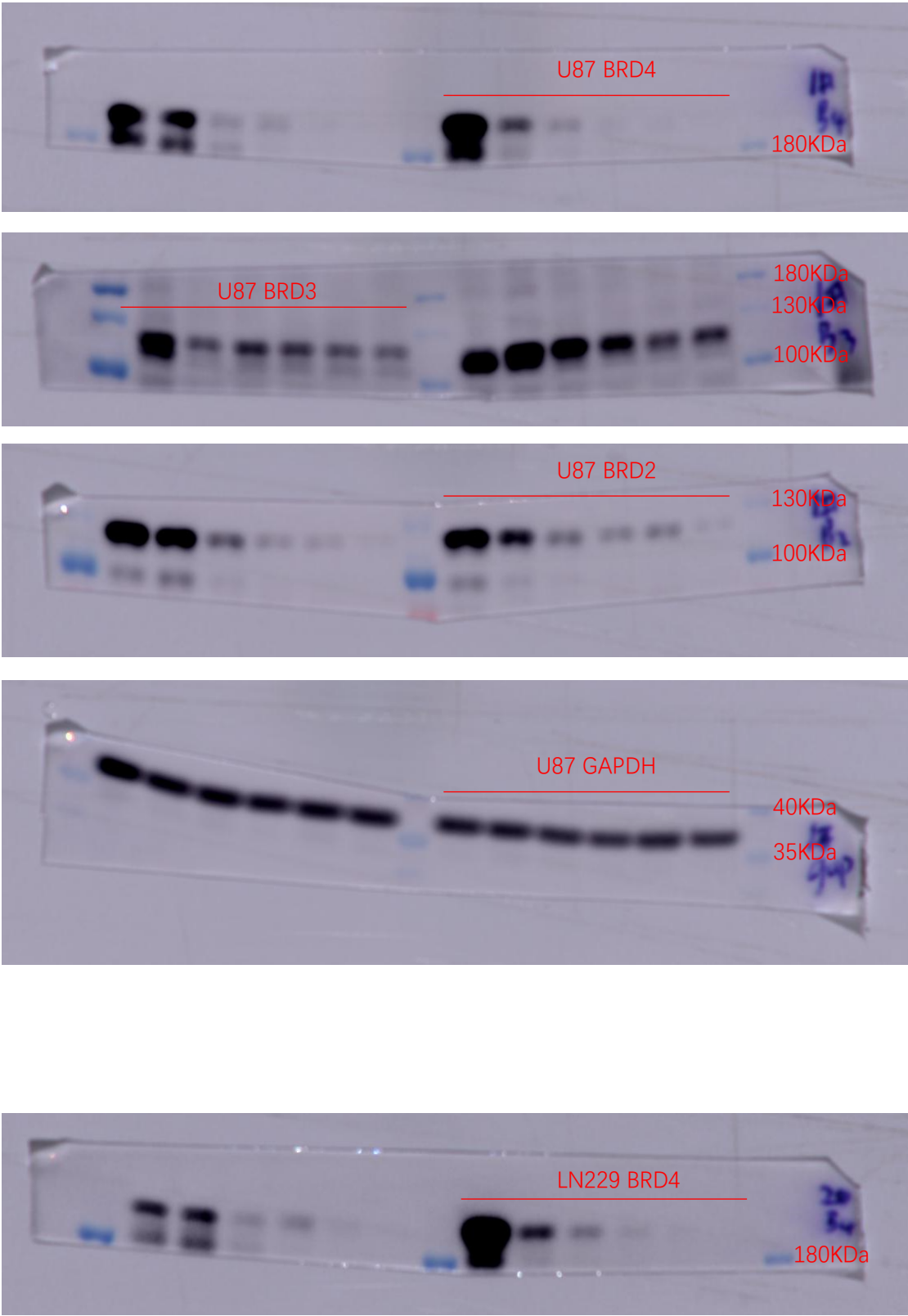

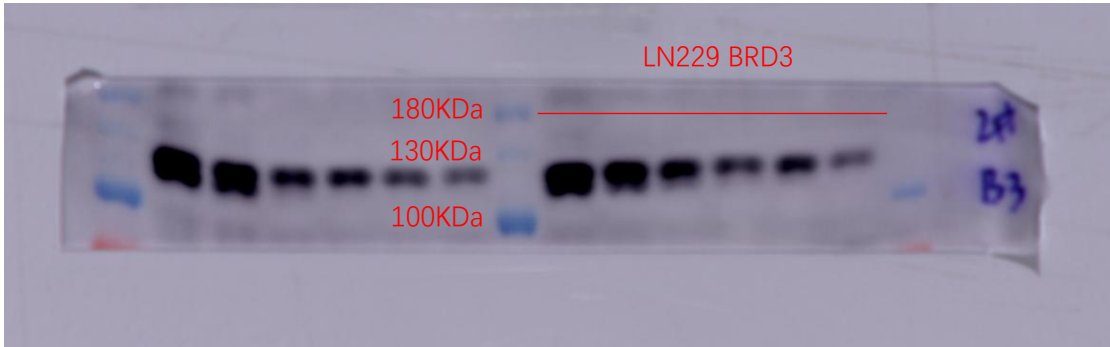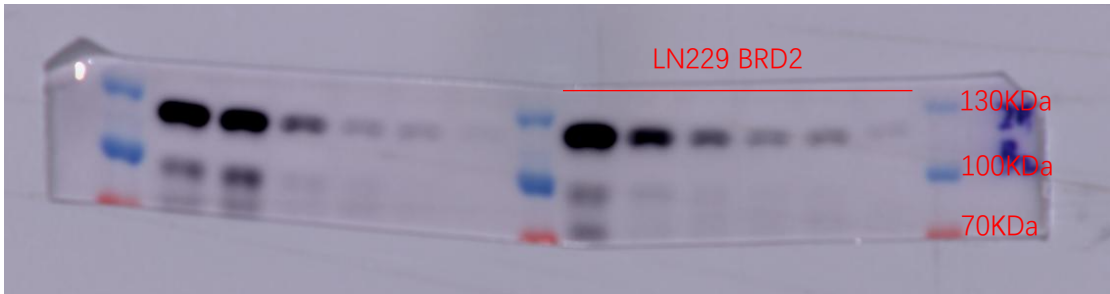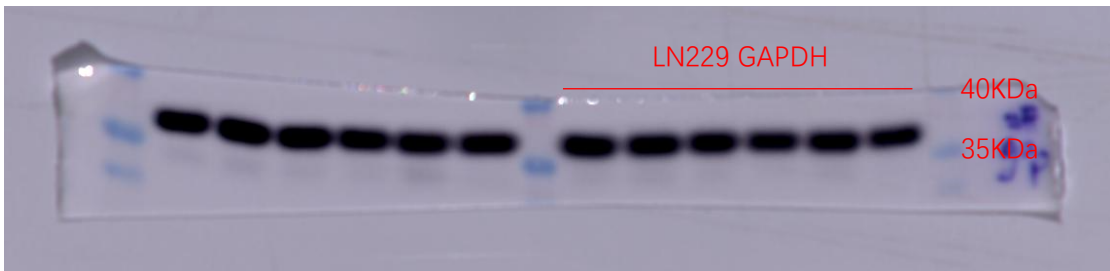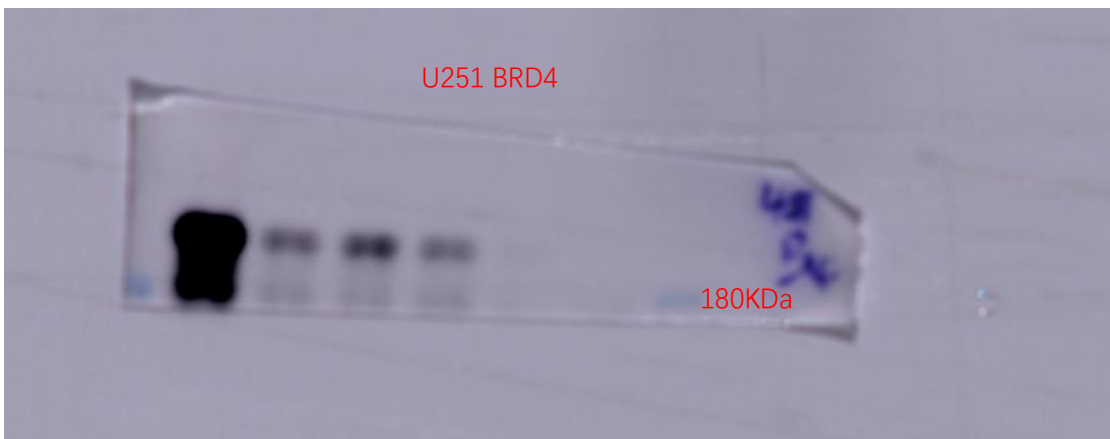

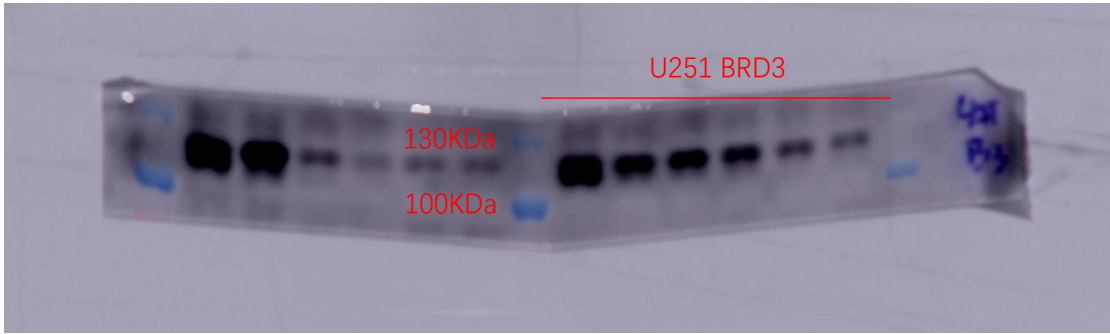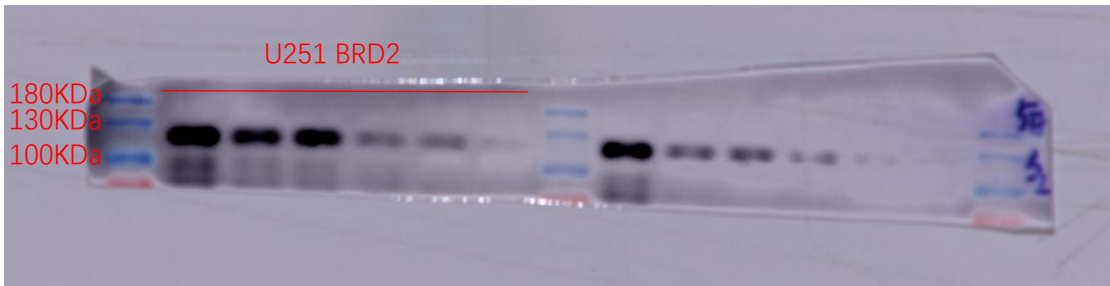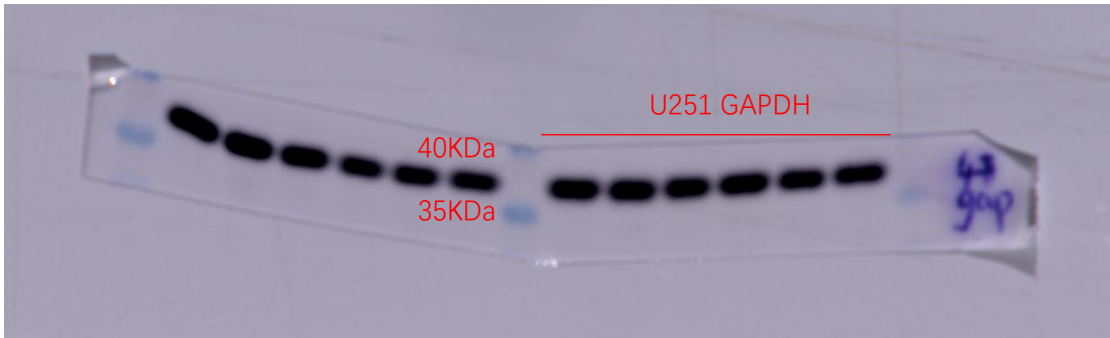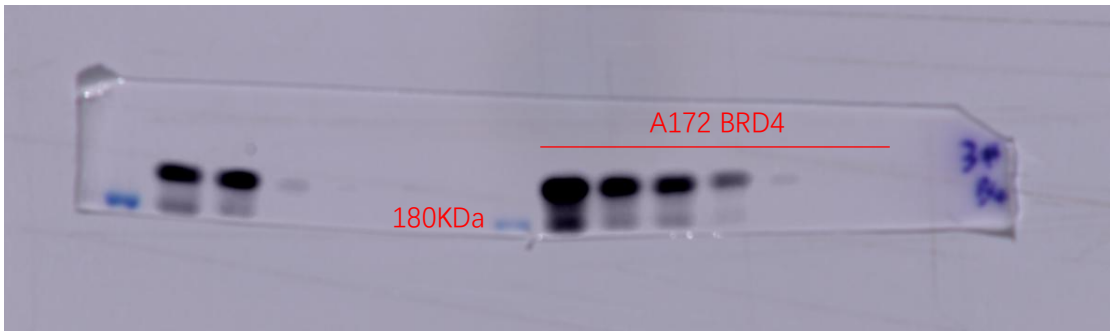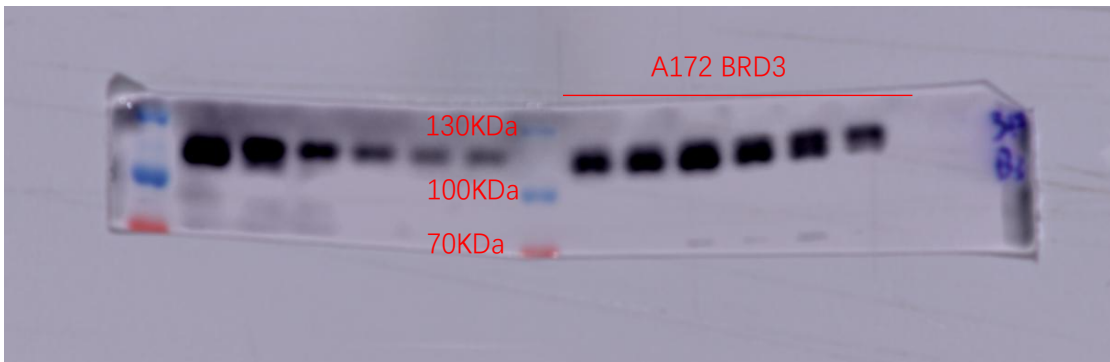

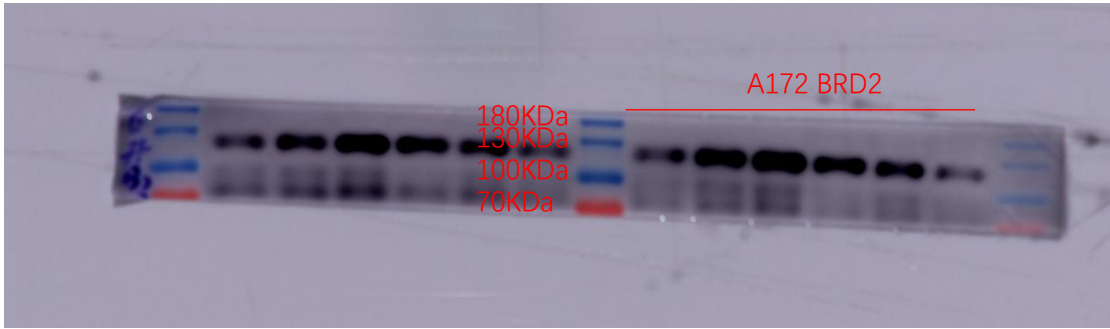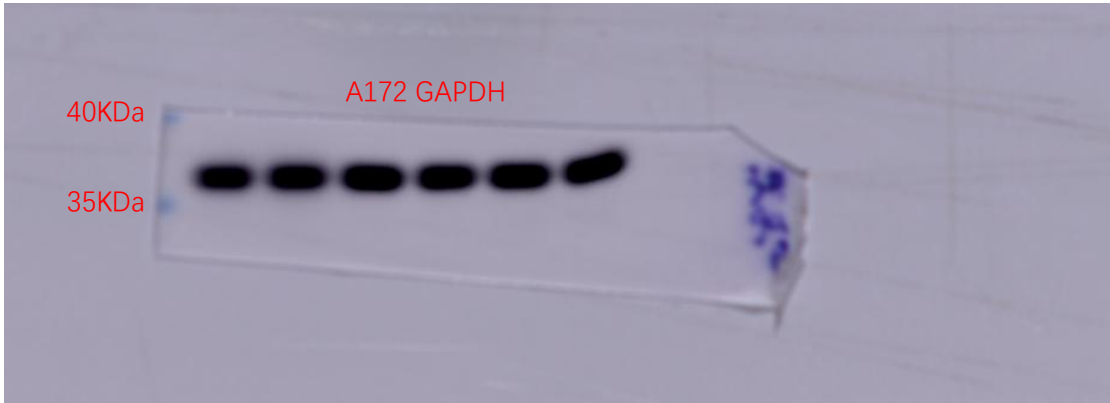

figure 3b

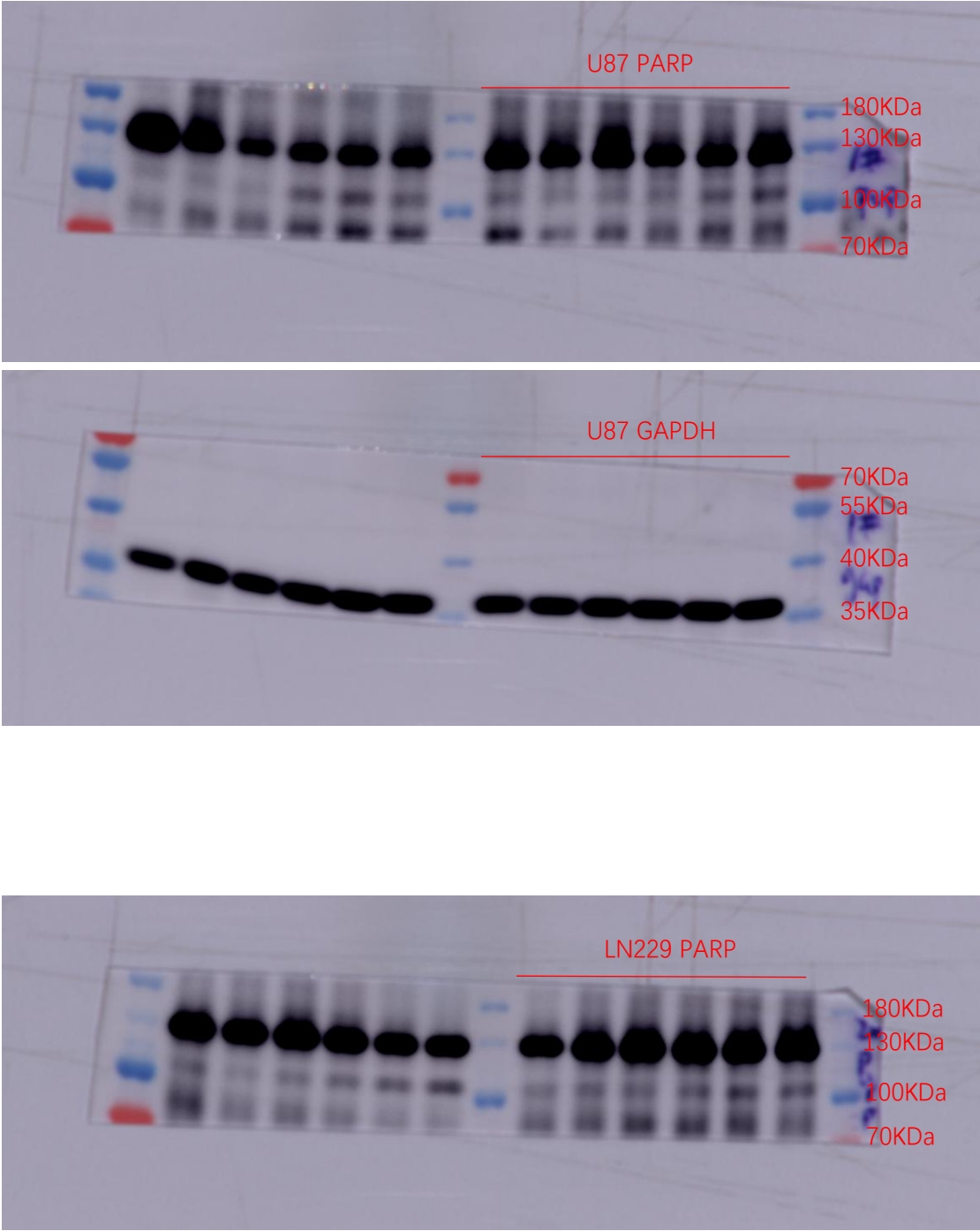

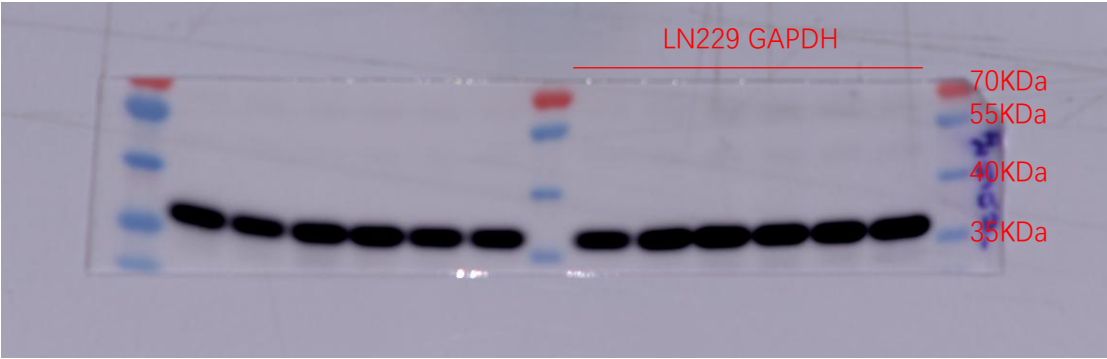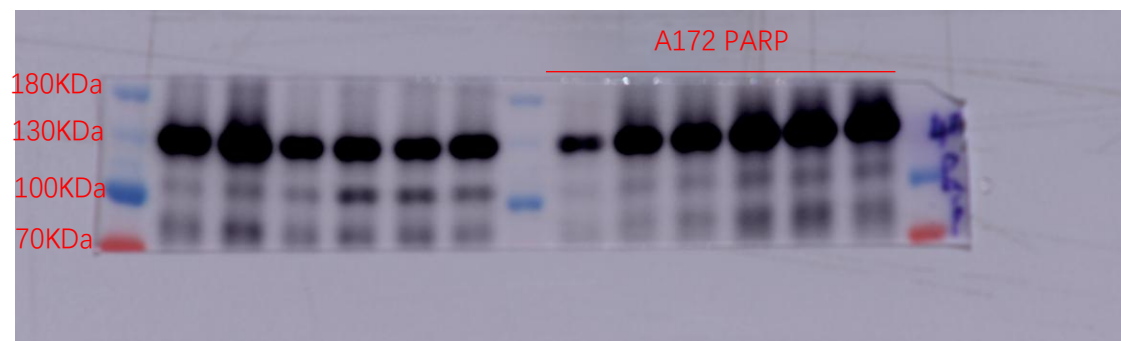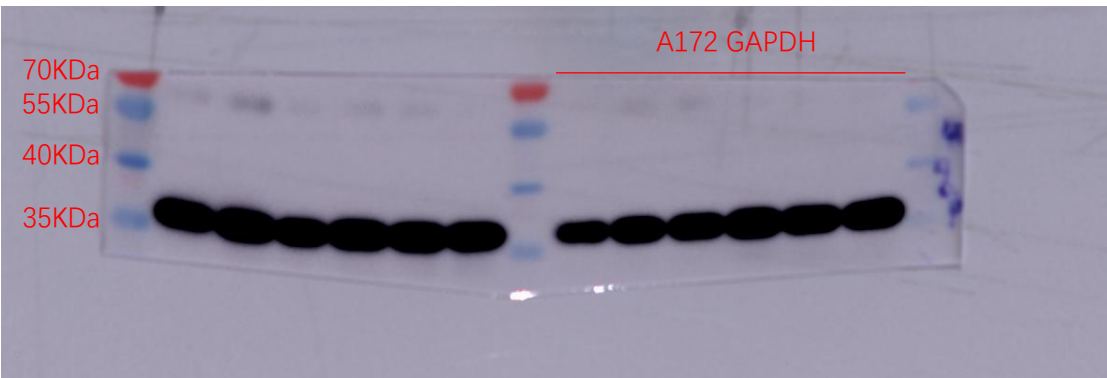

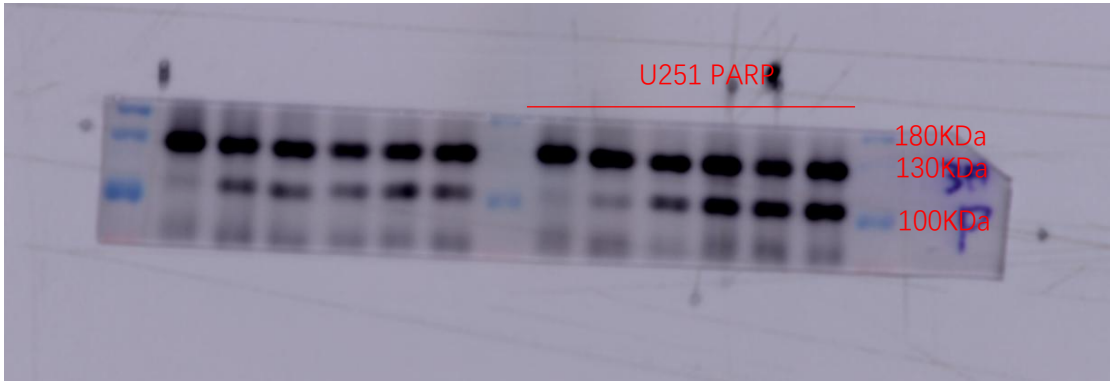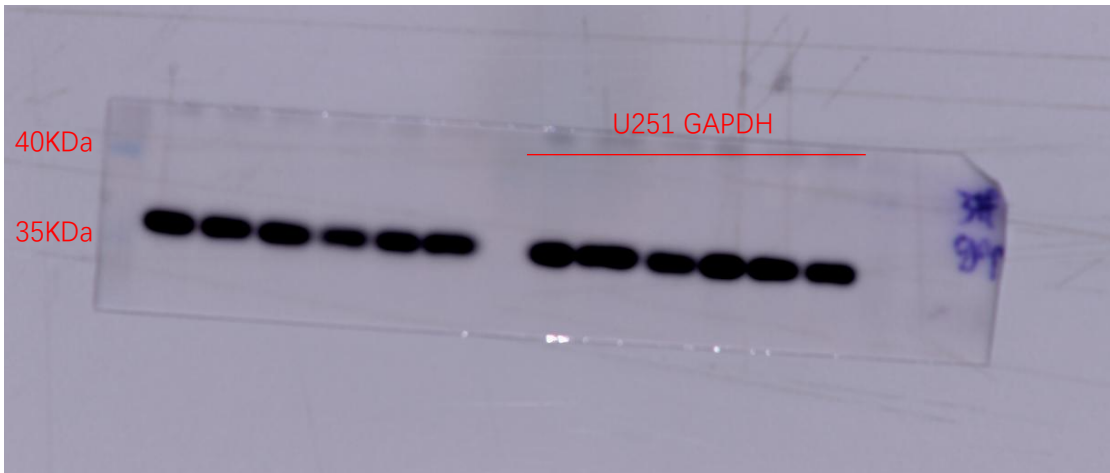

figure 4b

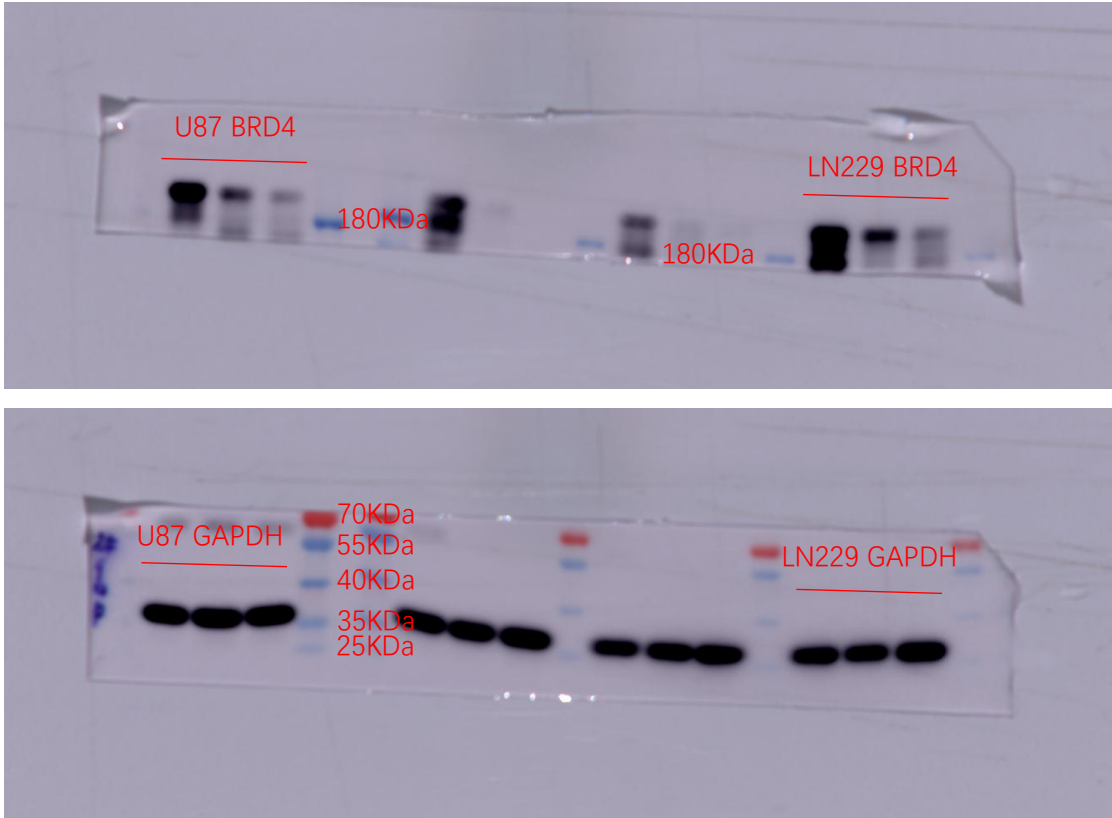

figure 4d

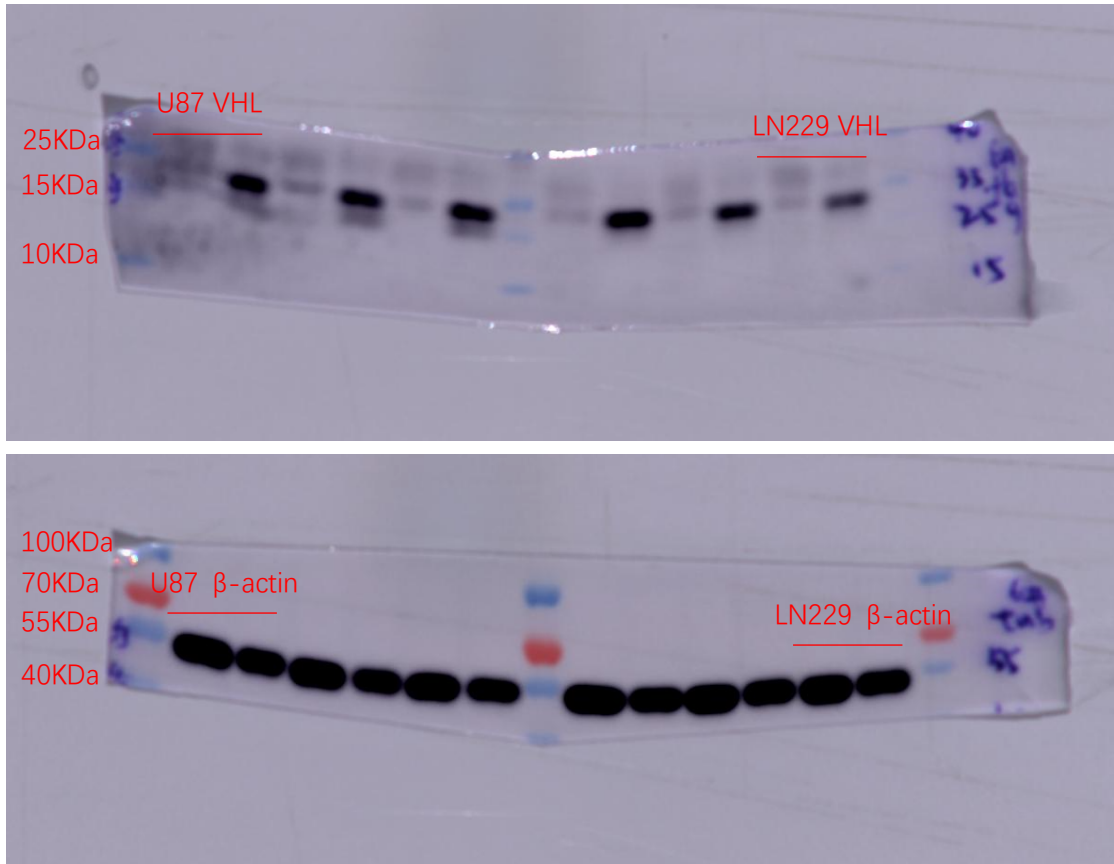

figure 4f

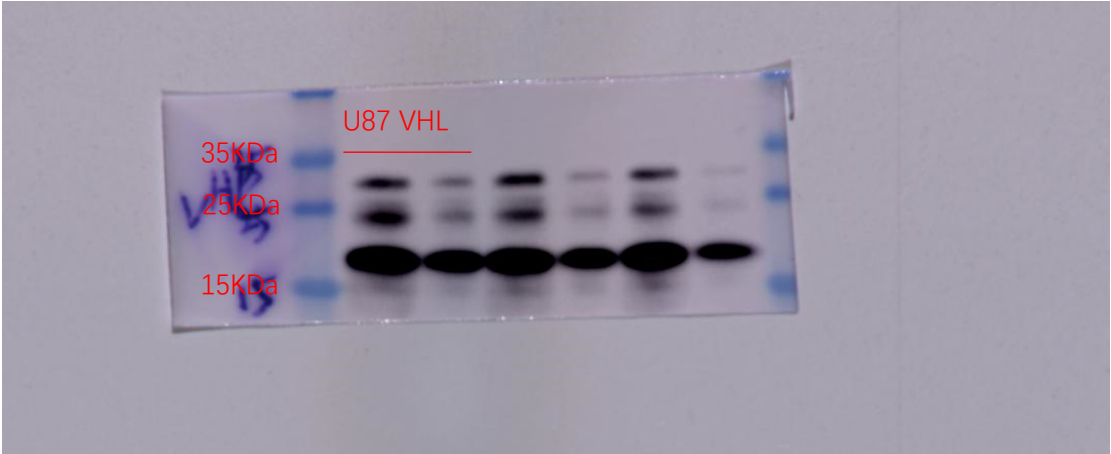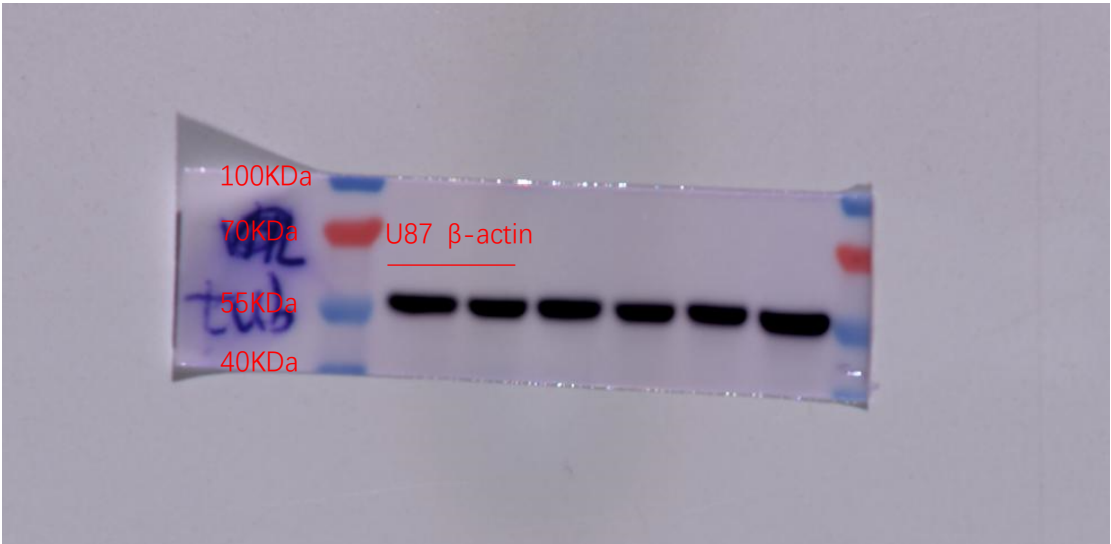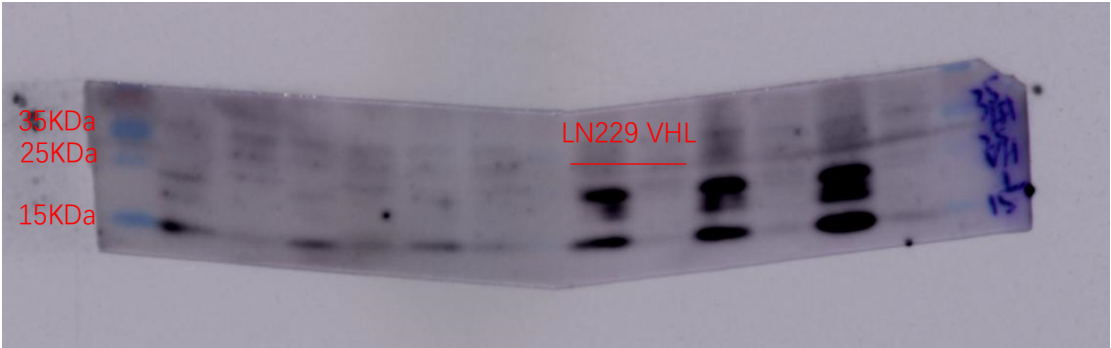

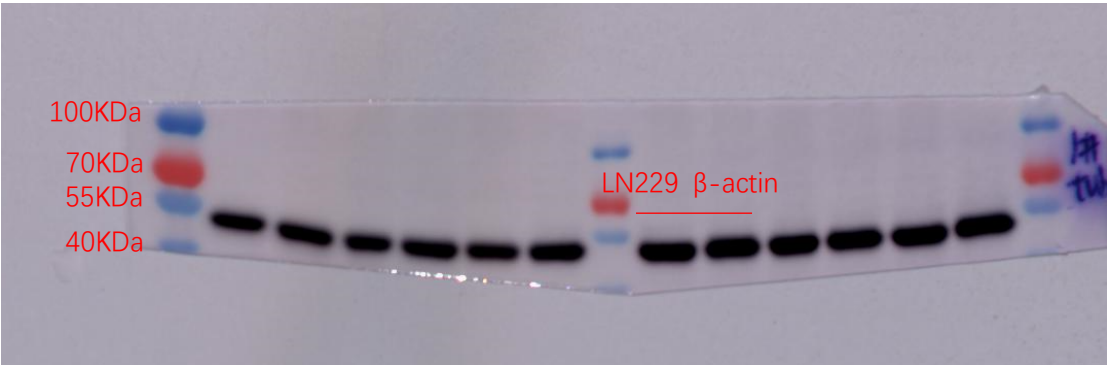

figure 5g

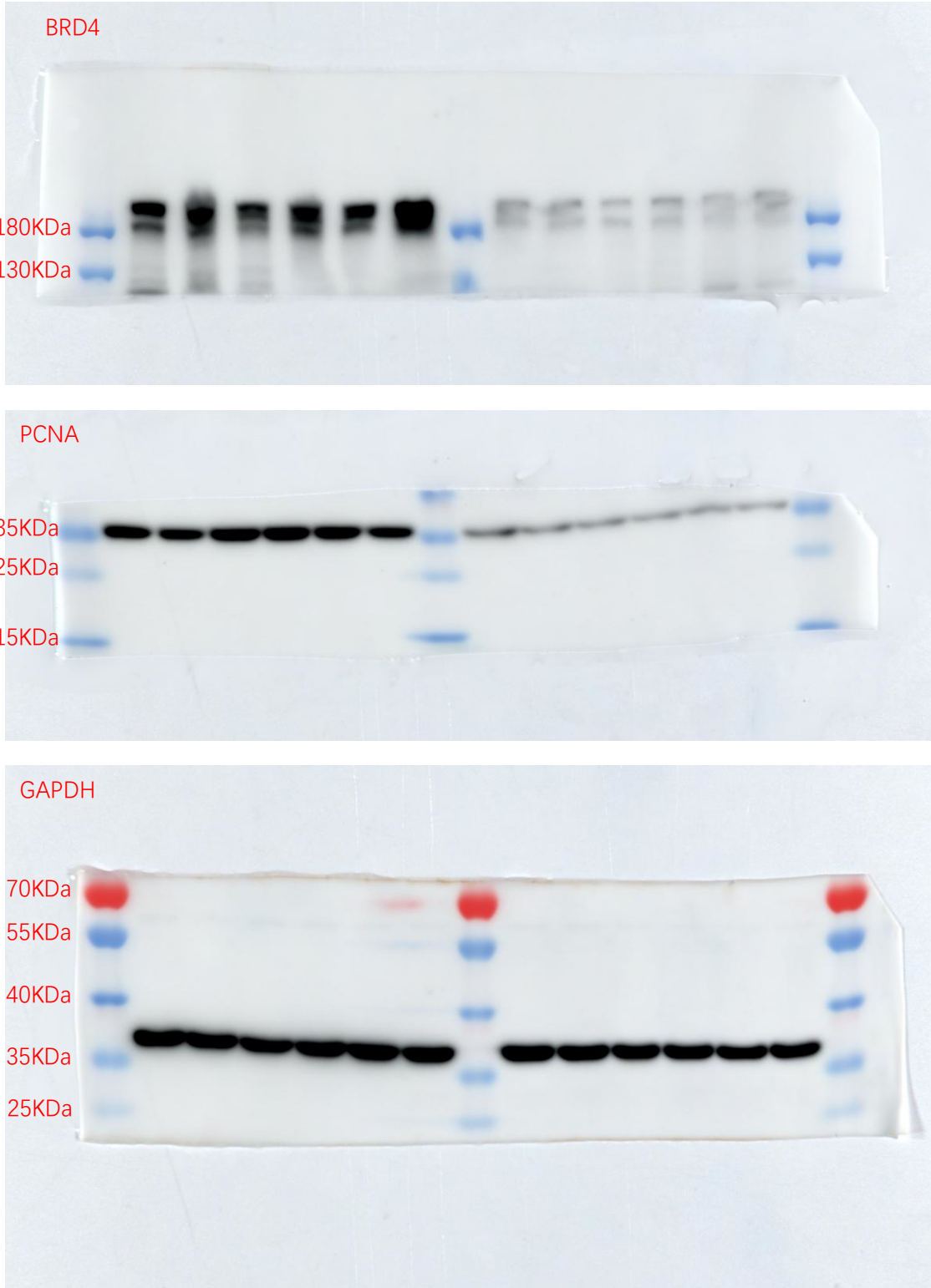

figure 7h

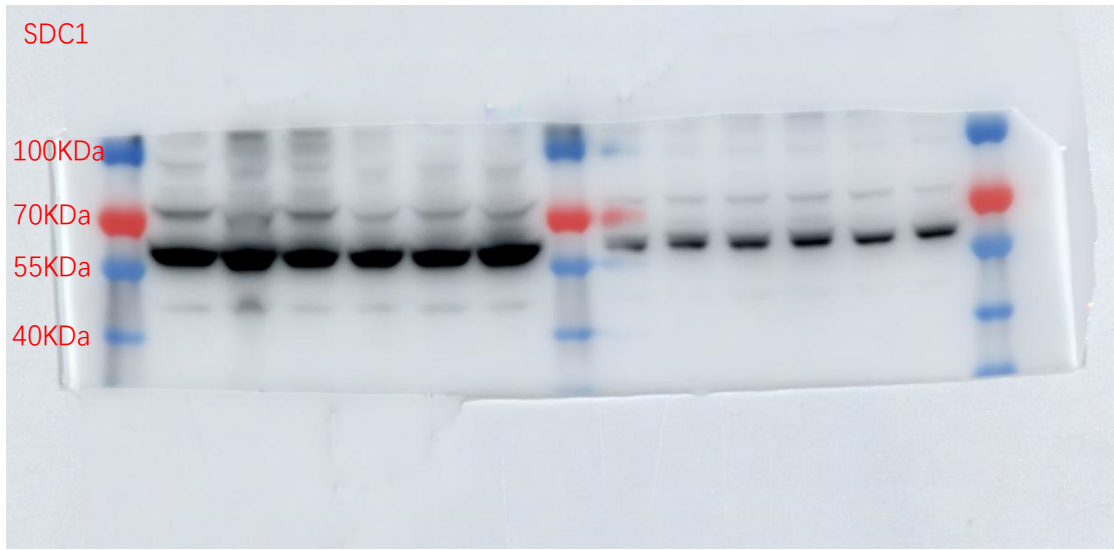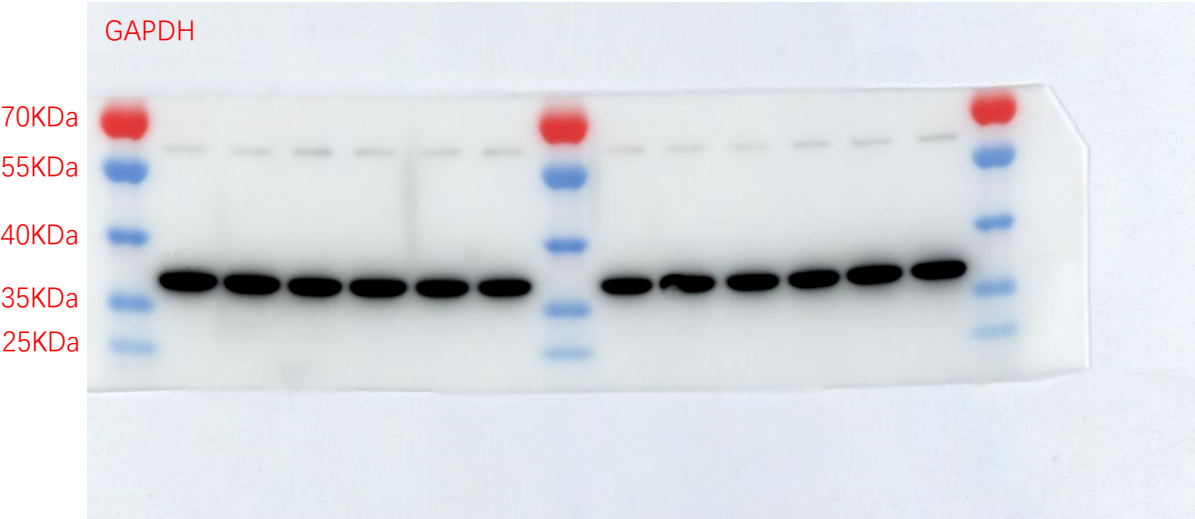

figure 7j

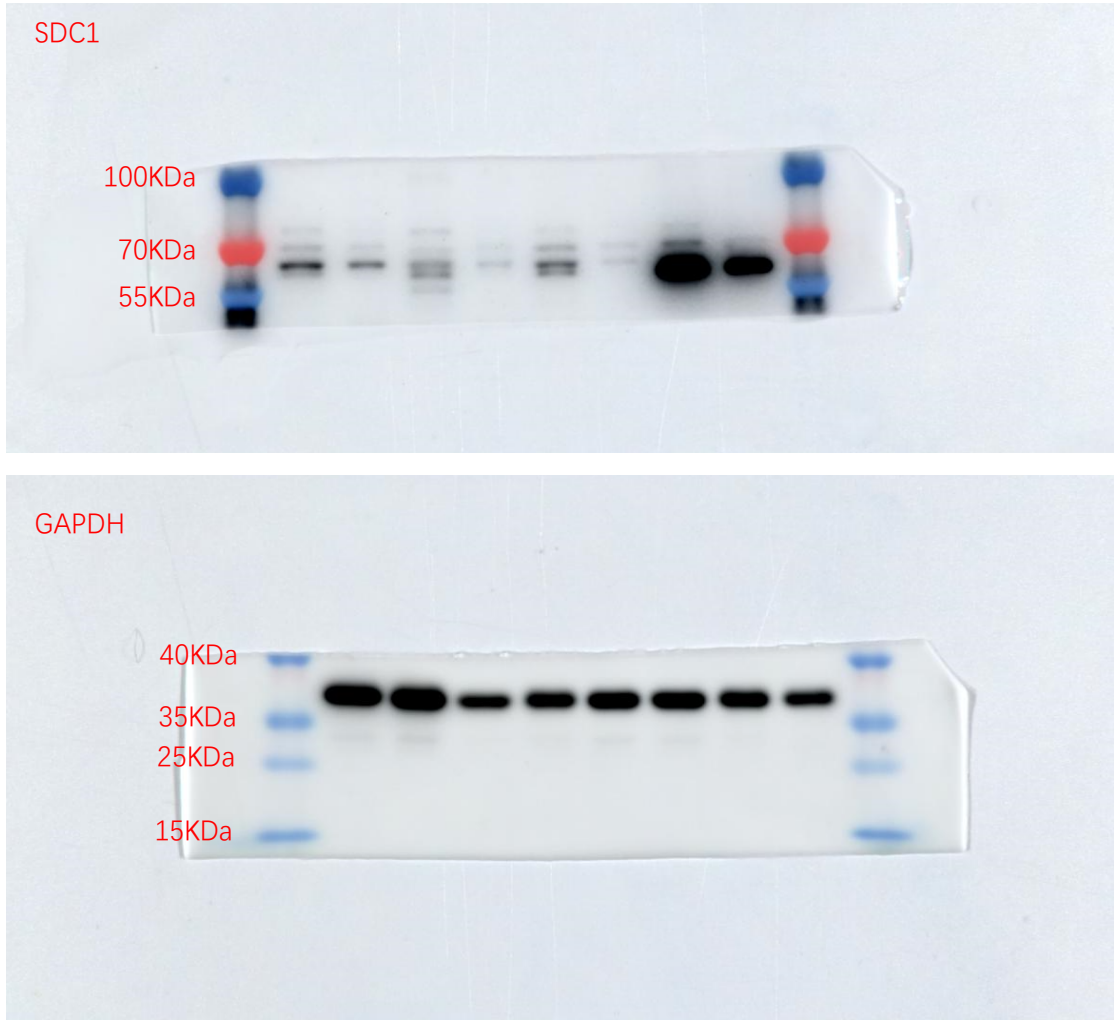

figure 8a

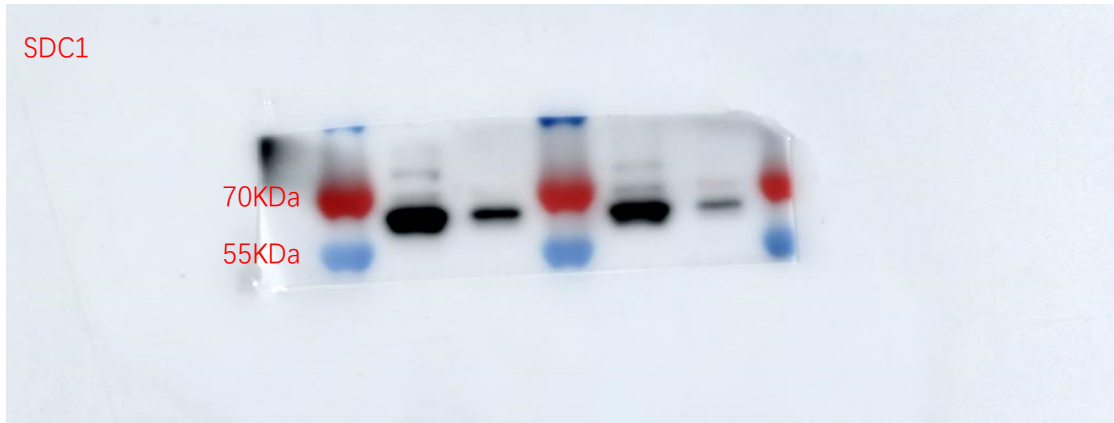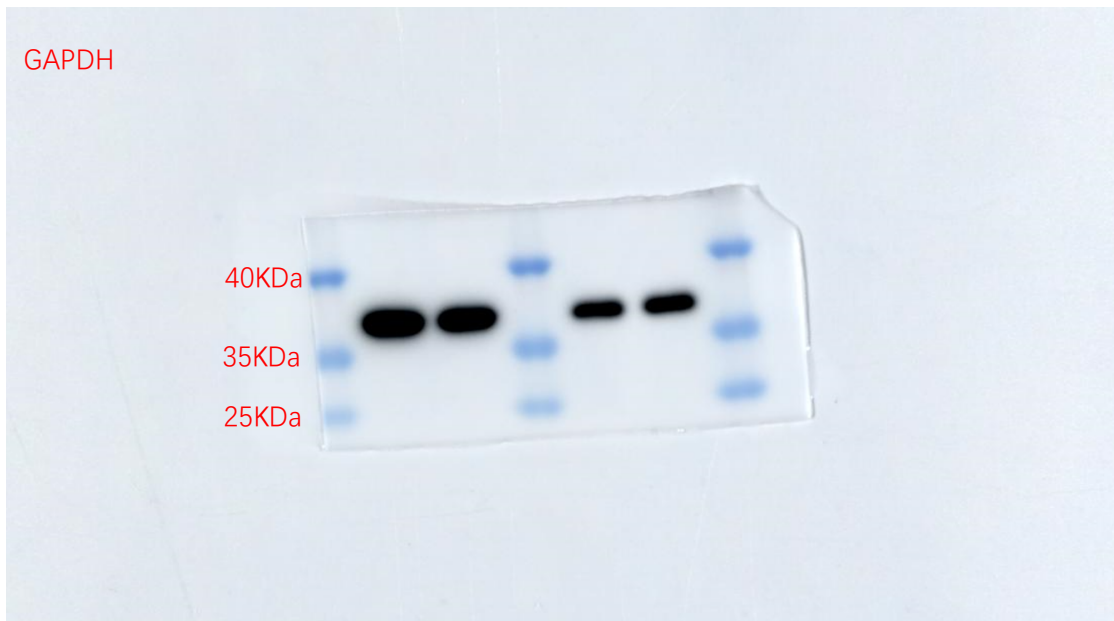

Supplement: Supplementary file 1 — Supplementary material 1. [file 12885_2024_11966_MOESM1_ESM.pdf]
